# Mitochondrial rRNA Methylation by Mettl15 Contributes to the Exercise and Learning Capability in Mice

**DOI:** 10.3390/ijms23116056

**Published:** 2022-05-27

**Authors:** Olga A. Averina, Ivan G. Laptev, Mariia A. Emelianova, Oleg A. Permyakov, Sofia S. Mariasina, Alyona I. Nikiforova, Vasily N. Manskikh, Olga O. Grigorieva, Anastasia K. Bolikhova, Gennady A. Kalabin, Olga A. Dontsova, Petr V. Sergiev

**Affiliations:** 1 Institute of Functional Genomics, Lomonosov Moscow State University, 119991 Moscow, Russia; averina.olga.msu@gmail.com (O.A.A.); norad_m@mail.ru (O.A.P.); sm1024sm@yandex.ru (S.S.M.); grig_forever@mail.ru (O.O.G.); 2 Belozersky Institute of Physico-Chemical Biology, Lomonosov Mosco16w State University, 119991 Moscow, Russia; whiteswan92@gmail.com (I.G.L.); manskikh@mail.ru (V.N.M.); olga.a.dontsova@gmail.com (O.A.D.); 3 Center of Life Sciences, Skolkovo Institute of Science and Technology, 121205 Moscow, Russia; mshep98@mail.ru; 4 Institute of Mitoengineering, Lomonosov Moscow State University, 119234 Moscow, Russia; rabio@mail.ru; 5 Faculty of Bioengineering and Bioinformatics, Lomonosov Moscow State University, 119234 Moscow, Russia; anastasia_b7@mail.ru; 6 Pharmacy Resource Center, Peoples’ Friendship University of Russia (RUDN), 117198 Moscow, Russia; kalabin-ga@rudn.ru; 7 Department of Chemistry, Lomonosov Moscow State University, 119991 Moscow, Russia; 8 Shemyakin-Ovchinnikov Institute of Bioorganic Chemistry, 117997 Moscow, Russia

**Keywords:** mitochondria, rRNA, methylation, knockout mice, translation

## Abstract

Mitochondrial translation is a unique relic of the symbiotic origin of the organelle. Alterations of its components cause a number of severe human diseases. Hereby we report a study of mice devoid of Mettl15 mitochondrial 12S rRNA methyltransferase, responsible for the formation of m^4^C839 residue (human numbering). Homozygous *Mettl15*^−/−^ mice appeared to be viable in contrast to other mitochondrial rRNA methyltransferase knockouts reported earlier. The phenotype of *Mettl15*^−/−^ mice is much milder than that of other mutants of mitochondrial translation apparatus. In agreement with the results obtained earlier for cell cultures with an inactivated *Mettl15* gene, we observed accumulation of the RbfA factor, normally associated with the precursor of the 28S subunit, in the 55S mitochondrial ribosome fraction of knockout mice. A lack of Mettl15 leads to a lower blood glucose level after physical exercise relative to that of the wild-type mice. *Mettl15*^−/−^ mice demonstrated suboptimal muscle performance and lower levels of Cox3 protein synthesized by mitoribosomes in the oxidative soleus muscles. Additionally, we detected decreased learning capabilities in the *Mettl15*^−/−^ knockout mice in the tests with both positive and negative reinforcement. Such properties make *Mettl15*^−/−^ knockout mice a suitable model for mild mitochondriopathies.

## 1. Introduction

Mitochondria, the powerhouses of eukaryotic cells, possess their own genome as a remnant of its endosymbiotic origin [1]. To synthesize 13 protein components of the oxidative phosphorylation (OXPHOS) complexes encoded in the mitochondrial genome, the organelle makes use of the specialized translation apparatus [2,3,4,5] inherited from the endosymbiotic proteobacteria. Although ribosomal and transfer RNAs necessary for the mitochondrial protein biosynthesis are encoded within the organelle, ribosomal proteins, translation, and biogenesis factors [6] are encoded by the nuclear DNA, synthesized by the cytosolic ribosomes, and imported into the mitochondria.

Mutations in genes’ coding for the components of mitochondrial protein biosynthesis apparatus cause a number of severe human diseases, such as mitochondrial encephalopathy, lactic acidosis, and stroke-like episodes (MELAS), caused by a mutation in the mitochondrial tRNA^Leu(UUR)^ gene [7] and myoclonic epilepsy with ragged red fibers (MERRF) caused by a mutation in the mitochondrial tRNA^Lys^ gene [8]. Later, a number of other mutations within the mitochondrial tRNA genes were found to be associated with similar pathologies [9]. Mutation A1555G in the mitochondrial 12S rRNA gene was found to cause sensorineural hearing loss exacerbated by aminoglycoside antibiotics [10]. A number of pathologies were found to be caused by mutations in nuclear genes’ coding for mitochondrial aminoacyl-tRNA synthetases, mitochondrial ribosomal proteins, as well as translation elongation and termination factors [11,12,13,14,15]. Mutations in genes’ coding for the proteins involved in mitochondrial ribosome biogenesis, such as ERAL1 [16], FASTKD2 [17], C1QBP/p32 [18], DHX30 [19], and GTPBP5 [20] are suggested as causative for human genetic disorders [15].

Mammalian mitochondrial rRNAs are modified at ten nucleotide residues [21,22,23], which are clustered in the functional centers of the small and large ribosomal subunits [24]. Mutations of the mitochondrial rRNA methyltransferase genes, such as TFB1M [25,26,27] and MRM2 [28], are also implicated in human diseases, such as maternally inherited deafness, diabetes, and MELAS-like syndrome. Although the list of mitochondrial rRNA modification enzymes was recently completed with the identification of the TRMT2B protein [29,30] as a 12S rRNA methyltransferase, the physiological impact of mitochondrial rRNA modifications at the level of an organism has been addressed for a limited number of related enzymes, such as TFB1M [31] and NSUN4 [32].

METTL15 is a mitochondrial rRNA methyltransferase, responsible for the 12S rRNA m^4^C839 modification [33,34,35]. Inactivation of the *METTL15* gene in various human [33,34] and murine [35] cell lines was accompanied by a variable degree of mitochondrial translation impairment ranging from almost negligible to severe, which could be attributed to the difference in cell lines used. To address the influence of the mitochondrial 12S rRNA modification at m^4^C839 residue (human numbering will be used throughout the manuscript for consistency with other publications) at the level of an organism, herein we describe the creation and study of a knockout mice line with homozygous inactivation of the *Mettl15* gene.

## 2. Results

### 2.1. Generation of Mettl15 Knockout Mice

**Figure 1:**
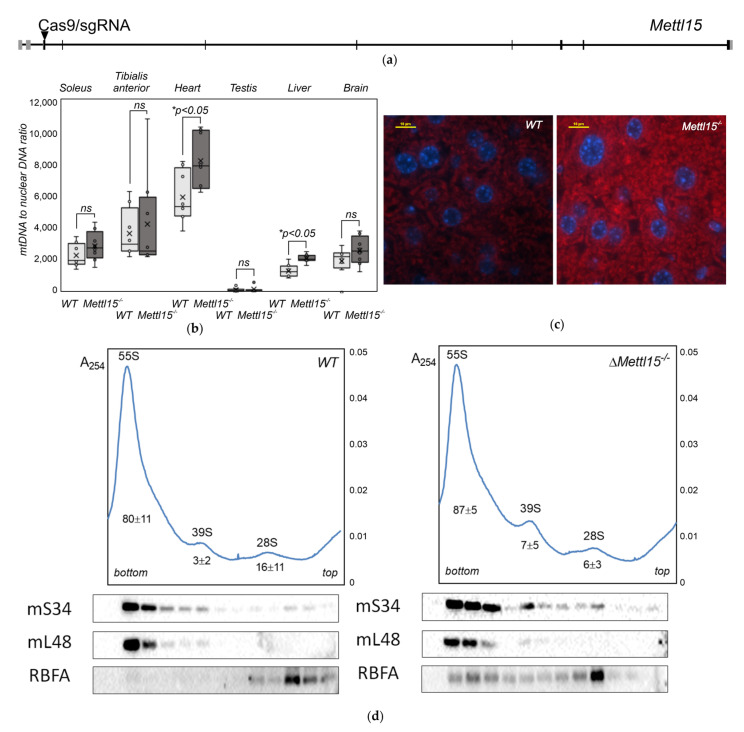
Influence of *Mettl15* gene inactivation on mitochondrial ribosomes in mice. (**a**) Scheme of *M. musculus Mettl15* gene structure with the site of CRISPR/Cas9 cleavage used to generate knockout. (**b**) Quantity of mtDNA relative to nuclear DNA assessed by qPCR in the wild-type (left box plot, light grey, *n* = 8) and *Mettl15^−/−^* knockout (right box plot, dark grey, *n* = 8) mice. Interquartile ranges are shown as solid bars and the entire data range by thin lines. Horizontal line corresponds to median while crossing to the average. Significance level calculated accordingly to the nonparametric Mann–Whitney U test is shown at *p* < 0.05. (**c**) Staining of liver mitochondria (red) by anti-VDAC1 antibodies for the wild type (left) and *Mettl15^−/−^* knockout (right) mice. Additional experiments and quantitation are shown in the Supplementary Materials Figure S2. (**d**) Sucrose-density gradient profiles of the liver mitochondrial extracts for the wild-type (left panel) and *Mettl15*^−/−^ knockout (right panel) mice. A_254_ is plotted on the upper panels and immunoblotting of the fractions on the lower panels. Antibodies used are indicated next to the panels. Peak quantitation averages on the basis of sucrose-density centrifugations of liver mitochondrial lysates of n = 8 mice on the basis of areas under curves are shown next to corresponding peaks. Additional experiments are shown in the Supplementary Materials Figure S6.

To inactivate the *Mettl15* gene in mice we applied a CRISPR/Cas9 system [36] to cleave the third (first coding) exon of the *Mettl15* gene (Figure 1a). To this end, a solution of the corresponding sgRNA obtained by in vitro transcription was mixed with *S. pyogenes* Cas9 coding mRNA and microinjected into zygotes of C57BL/6xCBA F1 mice. Following microinjection, the surviving zygotes were introduced to the oviducts of pseudopregnant mice. An inactivating two non-adjacent nucleotide deletion frameshift allele variant, hereafter referenced to as *Mettl15^−^* (Supplementary Materials Figure S1a,b), was found in the genotype of the progeny, which was used for further mating. Obtained heterozygous *Mettl15^+/−^* mice were backcrossed to a C57BL/6 background followed by mating to produce homozygous *Mettl15^−/−^* mice. Unlike knockouts for other mitochondrial rRNA methyltransferase genes [31,32], the homozygous *Mettl15^−/−^* mice were viable, borne at expected Mendelian ratios, and appeared normal.

The status of the 12S rRNA nucleotide C839 modification in the homozygous knockout mice was verified for several mice organs. To this end, the total RNA from the hearts, livers, and kidneys of four wild-type and four *Mettl15^−/−^* knockout mice were hybridized with the biotinylated oligonucleotide, complementary to the modification site. An excess of unhybridized RNA was digested by RNase T1, whereas the RNA fragment of interest was purified via immobilized streptavidin resin and subjected to RNase T1 hydrolysis followed by MALDI MS analysis (Supplementary Materials Figure S1c). As expected, we observed a complete lack of the 12S rRNA nucleotide C839 modification in all analyzed organs of the *Mettl15^−/−^* knockout mice, in contrast to a nearly complete modification in the corresponding organs of the wild-type mice. A complete lack of the nucleotide C839 modification in the 12S rRNA of *Mettl15^−/−^* knockout mice is accompanied by a substoichiometric modification of the neighboring nucleotide C837, in agreement with the observations obtained earlier for the *Mettl15* knockout cell lines [33,35].

### 2.2. Abundance of Mitochondria in Mettl15^−/−^ Knockout Mice

A malfunction of the mitochondrial rRNA modification machinery might lead to compensatory activation of the mitochondrial biogenesis and a consequent increase in the mtDNA copy number and the content of mitochondria in the cell [31,32]. To check whether *Mettl15* gene inactivation would result in an excessive mitochondria accumulation in mice tissues, we determined the mtDNA to nuclear DNA ratio in the cardiac and skeletal muscles, testis, liver, and brain via the qPCR method (Figure 1b). Although a tendency for an increase in the mtDNA copy number was observed in all analyzed tissues of the *Mettl15^−/−^* knockout mice except the glycolytic tibialis anterior muscles, it reached statistical significance in the liver and cardiac muscle.

Mitochondria were stained using the Altmann’s method [37] and with anti-VDAC1 antibodies in the samples of the liver and kidneys (Figure 1c, Supplementary Materials Figure S2). Although staining with the Altmann’s method revealed the tendency for increased mitochondrial content (Supplementary Materials Figure S2a,b,e) it does not reach statistical significance. Staining with anti-VDAC1 antibodies supported the same tendency (Supplementary Materials Figure S2c,f) for the kidney samples and demonstrated a significant (*p* < 0.05) excess of mitochondria in the liver of *Mettl15^−/−^* knockout mice (Figure 1c, Supplementary Materials Figure S2d,f).

To assess the possible pathological consequences of *Mettl15* gene inactivation we performed an assessment of the average body mass (Supplementary Materials Figure S3a) and temperature (Supplementary Materials Figure S3b) as well as a complete histopathology analysis of *Mettl15^−/−^* knockout mice and their wild-type littermates (Supplementary Materials Figure S3c).

No apparent differences that could be explained by *Mettl15* gene inactivation have been observed in the mass, thermogenesis, and histopathology analysis. The accelerated aging phenotype was described for mice carrying mutations in the components of the mitochondrial ribosome [38]. To assess the accumulation of senescent cells in the tissues of *Mettl15^−/−^* knockout mice we applied senescence-associated b-galactosidase activity tests. Sparse cells positive for b-galactosidase activity were observed in the kidneys of both the wild-type and *Mettl15^−/−^* knockout mice at 4 months of age (Supplementary Materials Figure S4a). The senescent cell density was almost equal for the four-month-old wild-type and *Mettl15*^−/−^ mice (Supplementary Materials Figure S4d). Other observed tissue samples, such as from the liver (Supplementary Materials Figure S4b), spleen, heart, brain, skeletal muscles, skin, and intestine (data not shown), were negative for b-galactosidase activity. However, an examination of a limited number of one-year-old wild-type (*n* = 2) and *Mettl15^−/−^* knockout mice (*n* = 2) allowed us to observe a nearly 2-fold difference in the number of senescent cells (Supplementary Materials Figure S4c) in the kidney slices. This remarkable tendency, albeit not conclusive due to the limited number of aged *Mettl15*^−/−^ mice, could be the subject of a separate study. To search for signs of mitochondrial myopathy, we looked for the red-stained pathological mitochondria using Gomori trichrome staining of frozen unfixed muscle sections (Supplementary Materials Figure S5). However, clear signs of pathological mitochondria were not observed in either of the samples.

### 2.3. Alteration in the Composition of the Mitochondrial Ribosomes in Mettl15^−/−^ Knockout Mice

Previously, we assessed an influence of the 12S rRNA modification at the nucleotide m^4^C839 on the proportion of mitochondrial 55S ribosomes and free 28S and 39S ribosomal subunits on the model of the murine NS0 cell line [35]. Although we found a very minute change in the ratio of the subunits and the ribosome caused by Mettl15 inactivation, the composition of mitochondrial ribosomal fractions was found to depend on the Mettl15 activity. Remarkably, RbfA, a mammalian mitoribosome assembly factor [39] homologous to the corresponding bacterial protein [40], was found to be associated with the 55S mitochondrial ribosome upon Mettl15 inactivation. Other groups observed variable degrees of mitoribosome assembly defects using human cell lines with an inactivated Mettl15 gene [33,34].

In order to check whether Mettl15 inactivation in mice would have an influence on mitoribosome association from the subunits and composition of mitochondrial ribosomes similar to those observed in cell lines, we prepared mitochondrial fractions from the liver samples of the *Mettl15^−/−^* knockout mice and their wild-type littermates. Mitochondrial lysates were subjected to ultracentrifugation through the sucrose-density gradients (Figure 1d, Supplementary Materials Figure S6a–h, upper panels). An influence of *Mettl15* knockout on the ratio of the ribosomal subunits and ribosomes in mice liver mitochondria was found to be minimal (Supplementary Materials Figure S6i), reminiscent of a similar observation made for the knockout murine cell line [35]. At the same time, immunoblotting of the sucrose-density gradient fractions (Figure 1d, Supplementary Materials Figure S6a–h, lower panels) confirmed an increased association of the RbfA assembly factor with the 55S ribosomes observed previously on a cell line model.

### 2.4. An Influence of Mettl15 Knockout on Gene Expression in Mice

**Figure 2:**
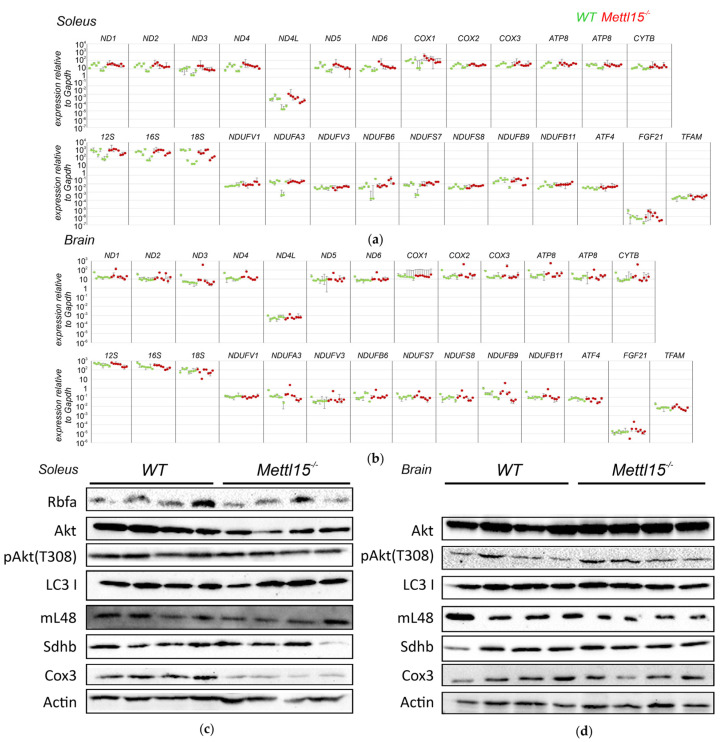
Influence of *Mettl15* gene inactivation on gene expression in mice. (**a**) RT qPCR quantification of the cytosolic and mitochondrial ribosomal RNA, mitochondrial mRNAs, and mRNAs of nuclear-encoded mitochondrial proteins as well as mRNAs reported to be overexpressed upon other mitochondrial deficiencies (designations shown above the lanes). Shown are values normalized to the level of *Gapdh* mRNA. Within the lane, each point corresponds to a wild-type (green dots, *n* = 8) or *Mettl15^−/−^* knockout (red dots, *n* = 8) animal (i.e., biological replicates). Error bars are used to illustrate the difference between technical replicates. RNA was extracted from oxidative muscle (soleus). Data for other tissues are shown in the Supplementary Materials Figure S7. (**b**) RT qPCR quantification of representative RNA amounts in the brains of the wild-type (green dots, *n* = 8) or *Mettl15^−/−^* knockout (red dots, *n* = 8) animals. Designations are similar to that of the panel (**a**). (**c**) Immunoblotting of the wild-type (left lanes, see additional experiments shown in the Supplementary Materials Figure S8, total *n* = 8) and *Mettl15*^−/−^ knockout (right three lanes, see additional experiments shown in the Supplementary Materials Figure S8, total *n* = 8) extracts from oxidative skeletal muscle (soleus). Antibodies used are indicated next to the panels. (**d**) Immunoblotting of the wild and *Mettl15*^−/−^ knockout extracts from the brain. Designations were similar to that of the panel (**b**).

In a number of studies, various mutations in the components of the mitochondrial translation apparatus were found to initiate a compensatory response manifested in increased transcription of the mitochondrial genome, an increase in the biogenesis of nuclear-encoded mitochondrial components, and boosting of the cytosolic translation efficiency via activation of mTOR signaling [41,42,43]. To check whether this is the case for the inactivation of *Mettl15* in mice, we analyzed the expression levels of the 16S and 12S mitochondrial rRNAs, all protein-coding genes of mitochondria, a number of nuclear genes of the mitochondrial proteins, and genes whose expression level was reported to be increased upon impairment of mitochondrial translation [42] in the heart, oxidative (soleus), and glycolytic (tibialis anterior) skeletal muscles, liver, testis, and brain. Cytosolic 18S rRNA and *Gapdh* mRNA were used as controls (Figure 2a,b, Supplementary Materials Figure S7). No significant alteration in gene expression was found to be associated with *Mettl15* gene knockout.

Additionally, quantities of the mitochondrial ribosomal protein Mrpl48, assembly factor RbfA, nuclear-encoded Sdhb, and mitochondrially encoded Cox3 protein components of the respiratory chain, as well as protein kinase Akt involved in mTOR signaling, its phosphorylated form, and autophagy factor LC3 have been assessed by immunoblotting of oxidative muscle (soleus) and brain lysates (Figure 2c,d, Supplementary Materials Figure S8). In line with the results of the RT qPCR analysis of the gene expression, we have detected neither a difference in the amounts of nuclear-encoded proteins nor the activities of mTOR signaling or autophagy between the wild-type and *Mettl15^−/−^* knockout mice. However, we observed a reduction in the amount of mitochondrially encoded Cox3 protein in the lysates of the oxidative soleus muscle. Since the Cox3 mRNA level was not affected by *Mettl15* gene inactivation (Figure 2a), it is likely that Cox3 protein level reduction is a result of a decrease in the mitochondrial translation efficiency caused by *Mettl15* inactivation. Such a difference, however, was not observed for the brain extracts.

### 2.5. An Influence of Mettl15 Knockout on the Physiology of Mice

**Figure 3:**
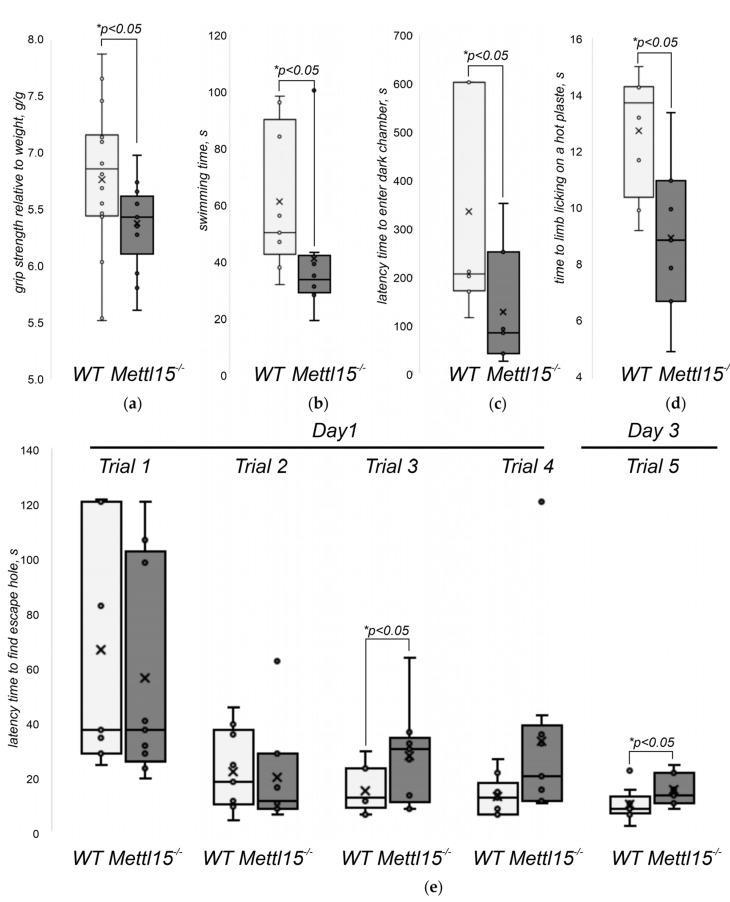
Influence of *Mettl15* gene inactivation on mice behavior. (**a**) Grip strength of the wild-type (left box plot, light grey, *n* = 16) and *Mettl15^−/−^* knockout (right box plot, dark grey, *n* = 13) normalized to the body weight. Interquartile ranges are shown as solid bars and the entire data range by thin lines. Horizontal lines correspond to median while crossing to the average. Significance level calculated accordingly to the nonparametric Mann–Whitney U test is shown at *p* < 0.05. (**b**) Forced swimming time in seconds with the weight load of 13% body weight of the wild-type (left box plot, light grey, *n* = 8) and *Mettl15^−/−^* knockout (right box plot, dark grey, *n* = 8). Designations are similar to the panel (**a**). (**c**) Learning efficiency with negative reinforcement. Shown is the latency time in seconds spent in the illuminated compartment prior to entering the dark compartment following the preceding training with electric foot shock in the dark compartment. Designations and number of animals similar to the panel (**b**). (**d**) Pain tolerance. Shown is the time spent on a heated plate (52–55 °C) before hind licking. Designations and number of animals similar to the panel (**b**). (**e**) Learning efficiency with positive reinforcement. Shown is the latency time in seconds spent in the illuminated box prior to entering the escape hole. The training trials 1–4 performed on the first day correspond to the bar groups 1–4. The test trial performed 48 h after learning corresponds to the rightmost group of bars. Designations and number of animals are similar to the panel (**b**).

The suboptimal function of mitochondria in general and mitochondrial protein biosynthesis in particular frequently leads to a decrease in the performance of systems that rely heavily on the oxidative phosphorylation, such as skeletal muscles, and a disturbance of the biochemical composition of body fluids is indicative of the increased usage of anaerobic glycolysis as an alternative to OXPHOS in energy production. To test whether this is the case for *Mettl15^−/−^* knockout mice we determined forelimb grip strength (Figure 3a) and forced floating time (Figure 3b) after 24 h of food deprivation. The performance of knockout mice in both tests was found to be significantly lower than that of the wild-type controls. Neurological manifestations were frequently found in mitochondrial diseases [44] and several mouse models with impaired mitochondrial functions demonstrated this [45,46,47]. We subjected the *Mettl15^−/−^* knockout mice to the open field test for general exploratory activity (Supplementary Materials Figure S9a) and preference for exploration of a new object (Supplementary Materials Figure S9b), and the light–dark box test to assess anxiety (Supplementary Materials Figure S9c) and learning (Figure 3c). Although the majority of tests revealed no difference between the wild-type and *Mettl15* knockout mice, we detected a significant difference between the groups in a learning test in a light–dark box with electric foot shock. In this test, mice were placed in a compartment with two boxes, a brightly illuminated one and a dark one, and were allowed to explore it freely. Mice tend to avoid illuminated boxes but were faced with a mild electric foot shock in the dark box. After placement in the home cage for 24 h, the mice were subjected to the same light–dark box to monitor their memories, which should suggest they avoid the dark box. To control for the possibility that *Mettl15^−/−^* knockout mice are more tolerant to the mild pain stimuli rather than their learning ability be compromised, we tested their response time latency in a hot plate test (Figure 3d). Apparently, mice with a Mettl15 deficiency are even more sensitive to pain than their wild-type littermates. Thus, we concluded that Mettl15 and hence mitoribosome modification contributes to the efficiency of learning or memory.

To explore further the learning deficit of the *Mettl15^−/−^* knockout mice, we used a memory test with positive rather than negative reinforcement. In the box maze test [48], mice were placed into the box with several dead-end appendices and an escape hole leading to a refuge. After initial free exploration of the maze, mice were subjected to four trials to find refuge. After 48 h, mice were subjected to a single trial to find refuge. The latency time before entering the escape hole is a readout (Figure 3e). As a result, we found that *Mettl15^−/−^* knockout mice performed worse in the box maze test spending significantly (*p* < 0.05) more time to recall the way to a known escape hole 2 days after training (Figure 3e, rightmost bars). This tendency was notable even during the training trials but reached statistical significance only for the third attempt (Figure 3e, third group of bars).

Patients suffering from a number of mitochondrial diseases frequently have lactic acidosis and a number of other differences in the plasma metabolites concentration [49]. The inactivation of genes related to the functioning of mitochondria in mice likewise results in plasma metabolite profile alterations [50]. To this end, we performed NMR-based metabolite profiling in the serum of the *Mettl15^−/−^* knockout mice and the corresponding control group fed *ad libitum* (Supplementary Materials Figure S10). The inactivation of the *Mettl15* gene appeared not to cause any significant difference in the metabolite profiles of mice under normal conditions.

**Figure 4:**
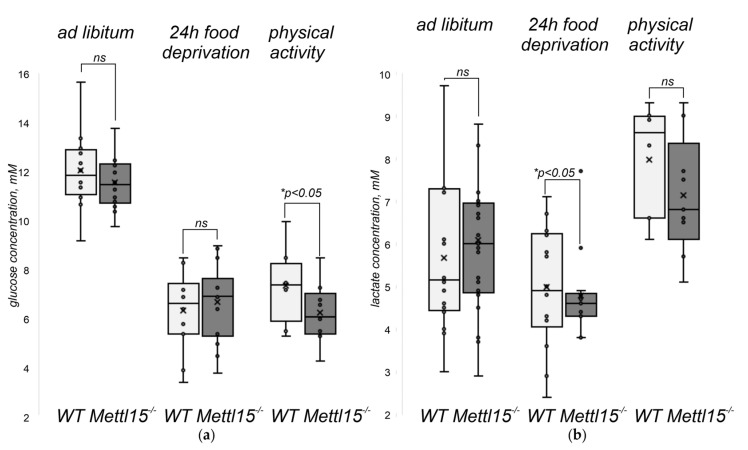
Influence of *Mettl15* gene inactivation on plasma glucose and lactate concentration. (**a**) Glucose concentration in the blood plasma of the wild-type (WT, left box plot, light grey) and *Mettl15^−/−^* knockout (*Mettl15^−/−^*, right box plot, dark grey) mice. Leftmost bars correspond to mice fed *ad libitum* (WT, *n* = 12; *Mettl15^−/−^*, *n* = 14). Central bars correspond to mice after 24 h food deprivation (WT, *n* = 14; *Mettl15^−/−^*, *n* = 14) and the rightmost bars to the mice after food deprivation and forced swimming (WT, *n* = 8; *Mettl15^−/−^*, *n* = 8). Interquartile ranges are shown as solid bars and the entire data range by thin lines. Horizontal lines correspond to median while crossing to the average. Significance level calculated accordingly to the nonparametric Mann–Whitney U test is shown at *p* < 0.05. (**b**) Lactate concentration in the blood plasma of the wild-type and *Mettl15^−/−^* knockout mice. Same conditions were compared: *ad libitum* nutrition (WT, *n* = 16; *Mettl15^−/−^*, *n* = 21), 24 h food deprivation (WT, *n* = 14; *Mettl15^−/−^*, *n* = 14) and following forced swimming (WT, *n* = 8; *Mettl15^−/−^*, *n* = 8). Designations similar to the panel (**a**).

In addition, we monitored the serum concentration of glucose (Figure 4a) and lactate (Figure 4b) of mice fed *ad libitum* (Figure 4a,b, leftmost bars), the same mice cohort after a 24 h food deprivation (Figure 4a,b, central bars), and the same mice after 24 h of food deprivation and forced swimming (Figure 4a,b, rightmost bars). As a result, we found that the fed *Mettl15^−/−^* mice demonstrate levels of plasma glucose and lactate concentrations nearly similar to the wild-type. After food deprivation, the plasma glucose concentrations dropped in both mice cohorts, whereas the lactate concentration remained nearly the same for the wild-type mice but decreased for the *Mettl15^−/−^* knockout mice to a level significantly lower than that of the wild-type. The intense physical exercise led to the elevation of the plasma lactate concentration for both the wild-type and knockout mice. Although the lactate concentration remained somewhat lower in the plasma of *Mettl15^−/−^* knockout mice, this difference was not significant. During this physical exercise, the glucose level of the wild-type mice increased, whereas that of the knockout mice decreased. As a result, the plasma glucose concentration following exercise was significantly lower for *Mettl15^−/−^* knockout mice.

Translation, as well as ribosome biogenesis, are energy-consuming processes, hence, even a moderate decrease in translation efficiency might lead to increased energy expenditure in mitochondria, which could be manifested in a lower reserve of energy left after a starvation period. Although the wild-type animals are likely to retain reserves of glycogen sufficient to supply glucose to the bloodstream in a period of high physical activity, *Mettl15^−/−^* knockout mice might be lacking it. To check for this possibility, we stained the samples of liver and skeletal muscle (gastrocnemius, the mixed-type muscle relying on both OXPHOS and glycolysis) for glycogen in the wild-type and *Mettl15^−/−^* knockout mice (Supplementary Materials Figure S11). In contrast to the expectation, no significant difference in the amount of glycogen was found between the wild-type and knockout mice. Thus, a somewhat lower concentration of glucose in the bloodstream of *Mettl15^−/−^* knockout mice following physical exercise is not a consequence of a systemic shortage of glycogen.

## 3. Discussion

Mitochondrial ribosomes are related to the ribosomes of their proteobacterial predecessors. Several modified nucleotides present in the mitochondrial rRNA are conserved between bacterial and mitochondrial ribosomes or universally conserved [24]. Three mitochondrial rRNA methyltransferases whose physiological significance has thus far been assessed on a knockout mice model belong to this category. Mitochondrial TFB1M methyltransferase [51] responsible for the formation of 12S rRNA dimethyladenosine residues m^6^_2_A936/7 is a homologue of bacterial KsgA protein [52] and Dim1/Dim1L protein [53] involved in the same modification of cytosolic ribosomes. Although an inactivation of KsgA in bacteria is not lethal, it leads to a ribosome biogenesis defect [54,55]; an inactivation of the *Dim1* gene responsible for the modification of the cytosolic ribosomes is lethal for yeast [53]; and an inactivation of the mitochondrial rRNA methyltransferase gene *Tfb1m* is lethal for mice [31]. Lethality is likewise associated with the inactivation of mice mitochondrial rRNA methyltransferase gene *Nsun4*, responsible for the formation of m^5^C841 in the 12S rRNA [32]. Deletion of its bacterial homologue *rsmF* [56], is not lethal but rather has a mild phenotype [55]. The essentiality of NSUN4 in mammals could be associated with an additional function of its complex with MTERF4 in the assembly of the large 39S mitochondrial ribosome subunit [32]. An inactivation of the *Mettl15* gene in mice reported here is not lethal and in contrast to other published knockouts of mitochondrial rRNA methyltransferase genes has a very mild phenotype. This is reminiscent of the mild phenotype of its bacterial ortholog *rsmH* knockout manifested only in a decrease in translation initiation fidelity [57] and the ability of the translation apparatus to cope with the burden of an exogenous gene expression [55].

We have observed neither an accumulation of ribosome assembly intermediates, which could be detected by a sucrose-gradient centrifugation, nor any defects in the formation of the mitochondrial 55S ribosomes in accordance with our earlier data obtained with the cell line with inactivated *Mettl15* [35]. However, we recapitulated the presence of the RbfA assembly factor [35] in the 55S ribosome fraction in *Mettl15^−/−^* knockout mice. It is likely that suboptimality of mitochondrial ribosome biogenesis caused by *Mettl15* inactivation is moderate and could have a physiological consequence only for tissues highly dependent on OXPHOS and under challenging conditions as will be discussed below. This statement is supported by the observation of the reduced level of Cox3 protein, synthesized by the mitoribosome, in the oxidative soleus muscle. Interestingly, the Cox3 protein amount in the brain is refractory to the *Mettl15* inactivation perhaps due to slower mitochondrial biogenesis and turnover.

Apart from the reported mice knockouts of *Tfb1m* [31] and *Nsun4* [32], the significance of mitochondrial protein biosynthesis components for mice physiology has been assessed for the mutations in genes’ coding for mitochondrial ribosomal proteins *Mrps5* [58], *Mrps34* [59], assembly factor *Ptcd1* [42], and mt-mRNA binding proteins *Slirp* [60] and *Taco1* [61], as well as translation factors *Mtif3* [62] and *Mtef4/Guf1* [41]. In many cases mutations in the assembly factors or ribosomal proteins lead to decreased stability of either 12S rRNA [31,59] or 16S rRNA [42] accompanied by an increase in transcription of rRNA whose stability was unaffected [31,32,42,60]; mitochondrially encoded mRNAs [31,32,42]; mRNAs coding for mitochondrial ribosomal proteins and transcription factors such as TFAM [32,59]; and a number of other nuclear genes such as *ATF4* and *FGF21,* whose expression is indicative of mitochondrial dysfunction [59]. At the posttranslational level, the excessive activation of mTOR signaling was likewise associated with suboptimal mitochondrial function [42,59]. Although we thoroughly assessed the expression of all protein-coding genes in the mitochondrial genome, 12S and 16S rRNA, and the indicative nuclear genes related to the mitochondria, we found that an inactivation of the *Mettl15* gene has a minor influence on gene expression in a representative range of tissues. Likewise, we could not detect a difference in the amounts of proteins related to mitochondrial function, mTOR signaling, and autophagy between the wild-type and *Mettl15* knockout mice. However, we observed the tendency for an increase in mtDNA content in the *Mettl15^−/−^* knockout mice tissues that was significant for the liver and heart. Increased mitochondrial content was corroborated by immunohistochemical staining of the liver mitochondria. These results are in line with the much milder effect of *Mettl15* gene knockout on the mitochondrial ribosome assembly and protein synthesis we observed in this study and the previous study on the knockout cell line [35].

Severe defects of the mitochondrial translation were reported to affect a number of tissues, such as the heart and skeletal muscles, where necrotic foci and centralized nuclei were observed [42,59,63], the liver where excessive lipid accumulation and steatosis were found [59], and the testis, where size decrease and spermatogenesis defects were noted [41]. As *Mettl15* knockout has a much milder influence on mitochondrial ribosome assembly, it was unsurprising to observe no significant differences in the histopathology of the knockout mice tissues from that of their wild-type littermates. More specific changes, such as an accumulation of senescent cells and damaged mitochondria, were not observed at a significant level in the young *Mettl15^−/−^* knockout mice. However, we observed an interesting tendency for excessive senescent cell accumulation in one-year-old mice kidneys, which could be an interesting subject for further study.

Mitochondrial dysfunction is idiosyncratically associated with lactic acidosis [12,13] as well as elevated alanine, creatine, and lactate/pyruvate ratio [49]. In accordance with the mild phenotype of *Mettl15* knockout at the molecular level, we observed no difference in the metabolite concentrations at the steady-state condition. However, challenging mice's physiological state by food deprivation and physical exercise allowed us to reveal a number of differences from the wild-type. Although the wild-type mice were found capable to elevate serum glucose levels upon forced swimming after 24 h of starvation, *Mettl15^−/−^* knockout mice demonstrated a small but significant decrease in the concentration of glucose under the same conditions. The lower glucose concentration could not be explained by lower glycogen storage, but perhaps by higher glucose expenditure due to lower efficiency of mitochondrial function. Starvation also led to a decrease in serum lactate concentration for *Mettl15^−/−^* knockout mice, and even after forced swimming the lactate concentration increase was less than that for the wild-type mice, although this difference was below statistical confidence (*p* > 0.05). A decrease in the glucose concentration associated with mitochondrial dysfunction provoked by starvation has been documented in a number of patients [64] including those who have alterations in the components of mitochondrial ribosomes [65]; in these cases, the molecular mechanisms leading to hypoglycemia have likewise remained enigmatic. Lower lactate levels in the serum of starved *Mettl15^−/−^* knockout mice could be a consequence of gluconeogenesis converting this metabolite into glucose to cope with glucose limitation.

Unsurprisingly, manifestations of the mitochondrial pathologies are associated with organs with high-energy demands, such as skeletal muscles and the brain [12]. *Mettl15^−/−^* knockout mice demonstrated a statistically significant force and performance reduction in the grip strength and forced swimming tests after a challenge of 24 h starvation. This effect might be explained by a lower energy production or higher expenditure; the latter being supported by a decreased plasma glucose concentration. A number of studies revealed a learning deficit caused by mutations in the components of the mitochondrial translation apparatus, such as Mrps34, Taco1, and Mrps5 [58,59,61]. Likewise, *Mettl15^−/−^* knockout mice demonstrated a significantly decreased learning capability. Moreover, we observed a learning deficit in the tests with either positive or negative reinforcement. The learning deficit in the Mrps5 mutant mice is attributed to the decrease in translation fidelity of the mitochondrial ribosomes [58]. A decrease in the fidelity of translation initiation was observed for a knockout of the bacterial ortholog of Mettl15, rRNA methyltransferase RsmH [57]. Whether phenotype manifestations of the *Mettl15* knockout are associated with mitochondrial mistranslation is a subject for further studies.

Mice lines with mutations in the genes coding for the components of mitochondrial translation apparatus are precious models for human mitochondrial diseases. Inactivation of other tested mitochondrial rRNA methyltransferase genes [31,32] were reported to be lethal, which speaks in favor of their importance but precludes their use as models of mitochondrial diseases. Obviously, inactivation of the *Mettl15* gene in mice has a way milder, yet significant muscular and neurological phenotype. This peculiarity paves the way for the application of *Mettl15^−/−^* knockout mice as a model for mild mitochondrial dysfunction.

## 4. Materials and Methods

### 4.1. Mettl15 Gene Inactivation and RNA Modification Analysis

For inactivation of the *M. musculus Mettl15* gene, we injected mouse zygotes with a mixture of 12 ng/uL sgRNA (target sequence GCATACTGAATCTAAAGCTG) mixed with 25 ng/uL mRNA coding for *S. pyogenesis* Cas9 (Thermo scientific). The genome-edited mice were generated as previously described [66]. All manipulations were conducted in compliance with protocol №182 approved by the Local Bioethics Commission of the Research Center “Institute of Mitoengineering of Moscow State University” LLC, (Moscow, Russia).

For the monitoring of the modification status of the 12S rRNA nucleotide C839, total RNA was isolated from the hearts, livers, and kidneys of four *Mettl15*^−/−^ mice and four mice of the control group by Trizol extraction. A 12S rRNA fragment was isolated by a previously published procedure [35] using 5′-biotinylated oligodeoxyribonucleotide (5′-[biotin]- TGTTAAGTTTAATTTAATTTGAGGAGGGTGACGGGCGGTG) complementary to the region of interest of the 12S rRNA and digested by RNase T1. Presence of the modification was analyzed by Ultraflex III BRUKER mass spectrometer equipped with a UV laser (Nd, 335 nm).

### 4.2. Tests with Live Mice

The experiments with live animals were performed on 8 wild-type animals and 8 *Mettl15*^−/−^ knockout mice. Statistical data analysis was performed using the nonparametric Mann–Whitney U test. The chosen significance level was *p* < 0.05.

The grip force test was done according to previously published procedure [67]. Each animal procedure was repeated 10 times with 15–30 s between attempts; five maximum values are averaged.

Forced swimming test [68,69,70,71,72,73] recording the animal's maximum swimming time represent physical performance capability. A mouse with 13% of body weight load fixed on the middle part of the tail was placed in water. The time until an animal’s refusal to swim is recorded. Mice were rescued alive from the water after the endpoint.

Novel object recognition test is designed to evaluate general exploratory activity and object recognition memory in rodents [74,75]. This methodology assesses the natural preference of rodents to explore novel objects [76]. Mice were allowed to explore two objects of the same shape and color for 5 min. The test was repeated the next day, one of the objects being replaced with a new one, different in color and shape from the initial one. The number of approximations, sniffing, and direct contacts of mice with the objects were recorded.

Passive avoidance test is fear-motivated test designed to evaluate memory in rodents. The test unit consists of a dark chamber with an electrode floor and a light chamber, separated by a wall with a sliding door. The mouse is placed in a brightly lit compartment, after which the door is opened and the latency period of passage to the compartment with the electrode floor is recorded. One day later, the animal is again placed in the illuminated compartment and the latency to cross through the opened gate between the compartments is assessed [77,78,79]. Memory performance is positively correlated with the latency of passage to the dark compartment on the second experimental day; the better the memory, the greater the latency.

Hot plate test is designed to assess nociception. The mouse is placed on a hot plate at a controlled temperature (52–55 °C) and the time until the first hind paw licking is recorded [80].

The light/dark transition test (LDT) is one of the most widely used tests to measure anxiety-like behavior in mice [81]. The LDT test is based on the innate aversion of rodents to brightly illuminated areas and on the spontaneous exploratory behavior of rodents in response to mild stressors, that is, novel environment and light [82]. The test unit consists of dark and light chambers. The latency of passage from the bright to the dark chamber was the studied research parameter.

Similar to the LDT test, the box-maze test [48] is based on rodents’ avoidance of brightly illuminated areas. The box-maze apparatus contains an illuminated central box with five holes leading to immediate dead ends and a single escape hole that is connected to the standard housing cage. Mice were placed into the illuminated box maze and allowed to explore it and behave freely for up to 2 min. The latency time to fully enter the escape hole is recorded. Mice are subjected to a total of four such trials, all on the same day. Over the course of the four trials, mice learn the location of the escape hole and exit the maze through it with progressively shorter escape latency. After 48 h, the procedure is repeated and mice are subjected to only one trial.

### 4.3. Histopathological Examination

The brain, heart, spleen, kidneys, liver, testis, lungs, tibialis anterior, and soleus muscle specimens were fixed with formalin, dehydrated and paraffin-embedded. Microtome sections with thickness of 3 µm were stained with hematoxylin and eosin. Images were observed and recorded using an Axiosope A1 microscope with a CCD-camera AxioCam MRc.5 (Carl Zeiss, Oberkochen, Germany).

To stain senescence-associated b-galactosidase positive senescent cells, we used the protocol for paraffin sections: organ samples were fixed with Baker’s formol-calcium solution for 24 h at +4 °C, washed in distilled water for 24 h, dehydrated in acetone, and embedded in paraffin [83,84]. Microtome sections with thickness of 5 µm were stained for 3 h with X-gal solution at suboptimal pH 6.0 and 37 °C as recommended [85] and counterstained with nuclear red. Number of senescent cells in full organ section was counted manually and averaged to 1 mm^2^ of section area with ImageJ software. To stain the mitochondria, the kidney and liver samples were fixed with Regaud fixative and stained according to classical Altmann’s method [37]. Area of mitochondria was estimated with ImageJ software on 3 photomicrographs (100×) of sections of renal tubules and liver. For study of myopathy lesions, unfixed cryotome sections with thickness of 5 µm were stained with Gomori trichrome [86].

Immunohistochemical analysis of mitochondrial content in the wild-type and *Mettl15^−/−^* knockout mice was done with formaldehyde-fixed paraffin-embedded slices of liver and kidneys. Staining was done with rabbit anti-VDAC1 antibodies (Abcam ab15895) and Cy5 conjugated goat anti-rabbit antibodies (Invitrogen, A10523). Microphotographs were taken by Nikon C2 microscope and analyzed by Nikon NIS-Elements software.

### 4.4. Sucrose-Gradient Profiling of Mitochondrial Ribosomes

Mitochondria and mitochondrial ribosomes were isolated by combining the methods described [87,88] with some changes. Mice were sacrificed by cervical dislocation and liver was homogenized in MIBSM buffer (50 mM HEPES-KOH, pH 7.5, 70 mM sucrose, 210 mM mannitol, 10 mM KCl, 1.5 mM MgCl_2_, 2 mM EDTA, 1 mM DTT, protease inhibitors). Lysate was clarified and mitochondria were pelleted by 7000× *g* 20 min centrifugation at +4 °C, resuspended and washed using the same procedure, and finally resuspended in 1 mL of SEM (20 mM HEPES-KOH, 250 mM sucrose, pH 7.5, 1 mM EDTA). Mitochondrial suspension was loaded on top of the step sucrose gradient (2 mL 60%, 4 mL 32%, 2 mL 23% and 2 mL 15% sucrose in 20 mM HEPES-KOH, pH 7.5, 1 mM EDTA) and centrifuged in SW41Ti rotor at 27,000 rpm for 1 h at +4 °C. Brown migrating band of mitochondria between 60% and 32% sucrose was collected and pelleted at 8000× *g* 10 min at +4 °C.

Mitoribosome profiles were obtained as described [35], fractionated into 0.5 mL fractions using ACTA purifier (GE Healthcare, Chicago, IL, USA) while monitoring absorbance at 254 nm. Proteins from gradient fractions were isolated by TCA/Deoxycholate precipitation as described [89], dissolved in 1× Laemmli buffer, and separated by SDS PAGE.

### 4.5. Western Blot Analysis

For Western blot analysis, mice organs were lysed in RIPA buffer with Halt Protease and Phosphatase Inhibitor Cocktail (ThermoFisher Scientific, Waltham, MA, USA) using Precellys Evolution homogenizer (Bertin Technologies, Montigny-le-Bretonneux, France). Protein concentration in cleared lysates was measured by Bradford assay and 30 µg of each lysate was used for Western blotting.

For detection of the proteins, the following antibodies were used: RBFA antibody (PA5-59587, Invitrogen, Carlsbad, CA, USA), MRPS34 antibody (PA5-59872, Invitrogen, Carlsbad, CA, USA), MRPL48 antibody (ab194826, Abcam, Waltham, MA, USA), AKT (9272, Cell Signaling Technology, Danvers, MA, USA), pAKT (Thr308) (4056, Cell Signaling Technology, Danvers, MA, USA), LC3A/B (PA1-16931, ThermoFisher Scientific, Waltham, MA, USA), SDHB (PA5-29843, ThermoFisher Scientific, Waltham, MA, USA), Cox3 (55082-1-AP, Proteintech, Rosemont, IL, USA), and Actin (ab8229, Abcam, Waltham, MA, USA).

### 4.6. Quantitative RT–PCR

Total RNA was extracted from mice tissue homogenates using QIAzol Lysis Reagent (Qiagen, Hilden, Germany). cDNA was synthesized from total RNA using Maxima First Strand cDNA Synthesis Kit for RT-qPCR (ThermoFisher Scientific, Waltham, MA, USA). The RT-PCR was performed on individual cDNAs by using SYBR^®^ Green PCR master mix in the CFX384 Touch Real-Time PCR System. The primer sequences are available in the Supplementary Materials (Supplementary Table S1). The mRNA expression was calculated by the 2^−ΔΔCT^ method and normalized to the expression of *Gapdh*.

Copy number of mtDNA was estimated by quantitative PCR as previously described [35].

### 4.7. Metabolome Analysis

For the analysis of lactate and glucose concentrations, blood samples were taken from the facial vein [90] and analyzed by a portable biochemical analyzer Accutrend Plus (Roche Diagnostics, Basel, Switzerland). For complete NMR analysis of metabolome, blood was collected by cardiac puncture [91] from animals anesthetized by isoflurane inhalation. Plasma was separated by centrifugation and used immediately for metabolite extraction by methanol and chloroform 1:1 mixture. Water layer was removed and dried. Before NMR measurements, the solid metabolites were dissolved in 50 mM sodium phosphate at pH 7.0, 0.107 mM d_6_-DSS, 0.13 mg/mL NaN_3_ in D_2_O. NMR spectra were acquired at 35 °C on a Bruker Avance 700 MHz NMR spectrometer equipped with a Z-axis-gradient 5 mm HCN Prodigy cryoprobe. One-dimensional ^1^H NMR spectra were acquired from each sample using the standard 1D NOESY-presat experiment. A total of 400 scans were accumulated for plasma samples. ^1^H,^13^C-HSQC spectra were measured for one sample from each group to confirm the metabolites assignments. All steps of 1D spectra processing and metabolite identification were performed using Chenomx NMR Suite program. ^1^H,^13^C-HSQC spectra were analysed in TopSpin program.

## Data Availability

Not applicable.

## Supplementary Materials

The following supporting information can be downloaded at: https://www.mdpi.com/article/10.3390/ijms23116056/s1.

## References

[1] Sagan L. (1993). On the Origin of Mitosing Cells. 1967. J. NIH Res..

[2] Christian B.E., Spremulli L.L. (2012). Mechanism of Protein Biosynthesis in Mammalian Mitochondria. Biochim. Biophys. Acta (BBA) Gene Regul. Mech..

[3] Ott M., Amunts A., Brown A. (2016). Organization and Regulation of Mitochondrial Protein Synthesis. Annu. Rev. Biochem..

[4] Mai N., Chrzanowska-Lightowlers Z.M.A., Lightowlers R.N. (2017). The Process of Mammalian Mitochondrial Protein Synthesis. Cell Tissue Res..

[5] Ayyub S.A., Varshney U. (2020). Translation Initiation in Mammalian Mitochondria- a Prokaryotic Perspective. RNA Biol..

[6] Lopez Sanchez M.I.G., Krüger A., Shiriaev D.I., Liu Y., Rorbach J. (2021). Human Mitoribosome Biogenesis and Its Emerging Links to Disease. Int. J. Mol. Sci..

[7] Goto Y., Nonaka I., Horai S. (1990). A Mutation in the TRNA(Leu)(UUR) Gene Associated with the MELAS Subgroup of Mitochondrial Encephalomyopathies. Nature.

[8] Shoffner J.M., Lott M.T., Lezza A.M., Seibel P., Ballinger S.W., Wallace D.C. (1990). Myoclonic Epilepsy and Ragged-Red Fiber Disease (MERRF) Is Associated with a Mitochondrial DNA TRNA(Lys) Mutation. Cell.

[9] Ruiz-Pesini E., Lott M.T., Procaccio V., Poole J.C., Brandon M.C., Mishmar D., Yi C., Kreuziger J., Baldi P., Wallace D.C. (2007). An Enhanced MITOMAP with a Global MtDNA Mutational Phylogeny. Nucleic Acids Res..

[10] Prezant T.R., Agapian J.V., Bohlman M.C., Bu X., Oztas S., Qiu W.Q., Arnos K.S., Cortopassi G.A., Jaber L., Rotter J.I. (1993). Mitochondrial Ribosomal RNA Mutation Associated with Both Antibiotic-Induced and Non-Syndromic Deafness. Nat. Genet..

[11] Rötig A. (2011). Human Diseases with Impaired Mitochondrial Protein Synthesis. Biochim. Biophys. Acta (BBA) Bioenerget..

[12] Vafai S.B., Mootha V.K. (2012). Mitochondrial Disorders as Windows into an Ancient Organelle. Nature.

[13] Boczonadi V., Horvath R. (2014). Mitochondria: Impaired Mitochondrial Translation in Human Disease. Int. J. Biochem. Cell Biol..

[14] Rudler D.L., Hughes L.A., Viola H.M., Hool L.C., Rackham O., Filipovska A. (2020). Fidelity and Coordination of Mitochondrial Protein Synthesis in Health and Disease. J. Physiol..

[15] Ferrari A., Del’Olio S., Barrientos A. (2021). The Diseased Mitoribosome. FEBS Lett..

[16] Chatzispyrou I.A., Alders M., Guerrero-Castillo S., Zapata Perez R., Haagmans M.A., Mouchiroud L., Koster J., Ofman R., Baas F., Waterham H.R. (2017). A Homozygous Missense Mutation in ERAL1, Encoding a Mitochondrial RRNA Chaperone, Causes Perrault Syndrome. Hum. Mol. Genet..

[17] Wei X., Du M., Li D., Wen S., Xie J., Li Y., Chen A., Zhang K., Xu P., Jia M. (2020). Mutations in FASTKD2 Are Associated with Mitochondrial Disease with Multi-OXPHOS Deficiency. Hum. Mutat..

[18] Feichtinger R.G., Oláhová M., Kishita Y., Garone C., Kremer L.S., Yagi M., Uchiumi T., Jourdain A.A., Thompson K., D’Souza A.R. (2017). Biallelic C1QBP Mutations Cause Severe Neonatal-, Childhood-, or Later-Onset Cardiomyopathy Associated with Combined Respiratory-Chain Deficiencies. Am. J. Hum. Genet..

[19] Lessel D., Schob C., Küry S., Reijnders M.R.F., Harel T., Eldomery M.K., Coban-Akdemir Z., Denecke J., Edvardson S., Colin E. (2017). De Novo Missense Mutations in DHX30 Impair Global Translation and Cause a Neurodevelopmental Disorder. Am. J. Hum. Genet..

[20] Solomon B.D., Pineda-Alvarez D.E., Hadley D.W., Keaton A.A., Agochukwu N.B., Raam M.S., Carlson-Donohoe H.E., Kamat A., Chandrasekharappa S.C. (2011). De Novo Deletion of Chromosome 20q13.33 in a Patient with Tracheo-Esophageal Fistula, Cardiac Defects and Genitourinary Anomalies Implicates GTPBP5 as a Candidate Gene. Birth Defects Res. A Clin. Mol. Teratol..

[21] Bohnsack M.T., Sloan K.E. (2018). The Mitochondrial Epitranscriptome: The Roles of RNA Modifications in Mitochondrial Translation and Human Disease. Cell. Mol. Life Sci..

[22] Laptev I., Dontsova O., Sergiev P. (2020). Epitranscriptomics of Mammalian Mitochondrial Ribosomal RNA. Cells.

[23] Lopez Sanchez M.I.G., Cipullo M., Gopalakrishna S., Khawaja A., Rorbach J. (2020). Methylation of Ribosomal RNA: A Mitochondrial Perspective. Front. Genet..

[24] Sergiev P.V., Aleksashin N.A., Chugunova A.A., Polikanov Y.S., Dontsova O.A. (2018). Structural and Evolutionary Insights into Ribosomal RNA Methylation. Nat. Chem. Biol..

[25] Bykhovskaya Y., Mengesha E., Wang D., Yang H., Estivill X., Shohat M., Fischel-Ghodsian N. (2004). Human Mitochondrial Transcription Factor B1 as a Modifier Gene for Hearing Loss Associated with the Mitochondrial A1555G Mutation. Mol. Genet. Metab..

[26] Cotney J., McKay S.E., Shadel G.S. (2009). Elucidation of Separate, but Collaborative Functions of the RRNA Methyltransferase-Related Human Mitochondrial Transcription Factors B1 and B2 in Mitochondrial Biogenesis Reveals New Insight into Maternally Inherited Deafness. Hum. Mol. Genet..

[27] Koeck T., Olsson A.H., Nitert M.D., Sharoyko V.V., Ladenvall C., Kotova O., Reiling E., Rönn T., Parikh H., Taneera J. (2011). A Common Variant in TFB1M Is Associated with Reduced Insulin Secretion and Increased Future Risk of Type 2 Diabetes. Cell Metab..

[28] Garone C., D’Souza A.R., Dallabona C., Lodi T., Rebelo-Guiomar P., Rorbach J., Donati M.A., Procopio E., Montomoli M., Guerrini R. (2017). Defective Mitochondrial RRNA Methyltransferase MRM2 Causes MELAS-like Clinical Syndrome. Hum. Mol. Genet..

[29] Laptev I., Shvetsova E., Levitskii S., Serebryakova M., Rubtsova M., Bogdanov A., Kamenski P., Sergiev P., Dontsova O. (2020). Mouse Trmt2B Protein Is a Dual Specific Mitochondrial Metyltransferase Responsible for M5U Formation in Both TRNA and RRNA. RNA Biol..

[30] Powell C.A., Minczuk M. (2020). TRMT2B Is Responsible for Both TRNA and RRNA M5U-Methylation in Human Mitochondria. RNA Biol..

[31] Metodiev M.D., Lesko N., Park C.B., Cámara Y., Shi Y., Wibom R., Hultenby K., Gustafsson C.M., Larsson N.-G. (2009). Methylation of 12S RRNA Is Necessary for in Vivo Stability of the Small Subunit of the Mammalian Mitochondrial Ribosome. Cell Metab..

[32] Metodiev M.D., Spåhr H., Loguercio Polosa P., Meharg C., Becker C., Altmueller J., Habermann B., Larsson N.-G., Ruzzenente B. (2014). NSUN4 Is a Dual Function Mitochondrial Protein Required for Both Methylation of 12S RRNA and Coordination of Mitoribosomal Assembly. PLoS Genet..

[33] Haute L.V., Hendrick A.G., D’Souza A.R., Powell C.A., Rebelo-Guiomar P., Harbour M.E., Ding S., Fearnley I.M., Andrews B., Minczuk M. (2019). METTL15 Introduces N4-Methylcytidine into Human Mitochondrial 12S RRNA and Is Required for Mitoribosome Biogenesis. Nucleic Acids Res..

[34] Chen H., Shi Z., Guo J., Chang K., Chen Q., Yao C.-H., Haigis M.C., Shi Y. (2020). The Human Mitochondrial 12S RRNA m ^4^C Methyltransferase METTL15 Is Required for Mitochondrial Function. J. Biol. Chem..

[35] Laptev I., Shvetsova E., Levitskii S., Serebryakova M., Rubtsova M., Zgoda V., Bogdanov A., Kamenski P., Sergiev P., Dontsova O. (2020). METTL15 Interacts with the Assembly Intermediate of Murine Mitochondrial Small Ribosomal Subunit to Form M4C840 12S RRNA Residue. Nucleic Acids Res..

[36] Ran F.A., Hsu P.D., Wright J., Agarwala V., Scott D.A., Zhang F. (2013). Genome Engineering Using the CRISPR-Cas9 System. Nat. Protoc..

[37] Romeis B. (1948). Mikroskopische Technik.

[38] Scherbakov D., Duscha S., Juskeviciene R., Restelli L., Frank S., Laczko E., Boettger E.C. (2020). Mitochondrial Misreading in Skeletal Muscle Accelerates Metabolic Aging and Confers Lipid Accumulation and Increased Inflammation. RNA.

[39] Rozanska A., Richter-Dennerlein R., Rorbach J., Gao F., Lewis R.J., Chrzanowska-Lightowlers Z.M., Lightowlers R.N. (2017). The Human RNA-Binding Protein RBFA Promotes the Maturation of the Mitochondrial Ribosome. Biochem. J..

[40] Xia B., Ke H., Shinde U., Inouye M. (2003). The Role of RbfA in 16S RRNA Processing and Cell Growth at Low Temperature in Escherichia Coli. J. Mol. Biol..

[41] Gao Y., Bai X., Zhang D., Han C., Yuan J., Liu W., Cao X., Chen Z., Shangguan F., Zhu Z. (2016). Mammalian Elongation Factor 4 Regulates Mitochondrial Translation Essential for Spermatogenesis. Nat. Struct. Mol. Biol..

[42] Perks K.L., Rossetti G., Kuznetsova I., Hughes L.A., Ermer J.A., Ferreira N., Busch J.D., Rudler D.L., Spahr H., Schöndorf T. (2018). PTCD1 Is Required for 16S RRNA Maturation Complex Stability and Mitochondrial Ribosome Assembly. Cell Rep..

[43] Ferreira N., Perks K.L., Rossetti G., Rudler D.L., Hughes L.A., Ermer J.A., Scott L.H., Kuznetsova I., Richman T.R., Narayana V.K. (2019). Stress Signaling and Cellular Proliferation Reverse the Effects of Mitochondrial Mistranslation. EMBO J..

[44] McFarland R., Taylor R.W., Turnbull D.M. (2010). A Neurological Perspective on Mitochondrial Disease. Lancet Neurol..

[45] Sörensen L., Ekstrand M., Silva, José P., Lindqvist E., Xu B., Rustin P., Olson L., Larsson N.-G. (2001). Late-Onset Corticohippocampal Neurodepletion Attributable to Catastrophic Failure of Oxidative Phosphorylation in MILON Mice. J. Neurosci..

[46] Weeber E.J., Levy M., Sampson M.J., Anflous K., Armstrong D.L., Brown S.E., Sweatt J.D., Craigen W.J. (2002). The Role of Mitochondrial Porins and the Permeability Transition Pore in Learning and Synaptic Plasticity. J. Biol. Chem..

[47] Aloni E., Ruggiero A., Gross A., Segal M. (2018). Learning Deficits in Adult Mitochondria Carrier Homolog 2 Forebrain Knockout Mouse. Neuroscience.

[48] Darvas M., Mukherjee K., Lee A., Ladiges W. (2020). A Novel One-Day Learning Procedure for Mice. Curr. Protoc. Mouse Biol..

[49] Shaham O., Slate N.G., Goldberger O., Xu Q., Ramanathan A., Souza A.L., Clish C.B., Sims K.B., Mootha V.K. (2010). A Plasma Signature of Human Mitochondrial Disease Revealed through Metabolic Profiling of Spent Media from Cultured Muscle Cells. Proc. Natl. Acad. Sci. USA.

[50] Graham B.H., Waymire K.G., Cottrell B., Trounce I.A., MacGregor G.R., Wallace D.C. (1997). A Mouse Model for Mitochondrial Myopathy and Cardiomyopathy Resulting from a Deficiency in the Heart/Muscle Isoform of the Adenine Nucleotide Translocator. Nat. Genet..

[51] Seidel-Rogol B.L., McCulloch V., Shadel G.S. (2003). Human Mitochondrial Transcription Factor B1 Methylates Ribosomal RNA at a Conserved Stem-Loop. Nat. Genet..

[52] Poldermans B., Roza L., Van Knippenberg P.H. (1979). Studies on the Function of Two Adjacent N6,N6-Dimethyladenosines near the 3’ End of 16 S Ribosomal RNA of Escherichia Coli. III. Purification and Properties of the Methylating Enzyme and Methylase-30 S Interactions. J. Biol. Chem..

[53] Lafontaine D., Vandenhaute J., Tollervey D. (1995). The 18S RRNA Dimethylase Dim1p Is Required for Pre-Ribosomal RNA Processing in Yeast. Genes Dev..

[54] Connolly K., Rife J.P., Culver G. (2008). Mechanistic Insight into the Ribosome Biogenesis Functions of the Ancient Protein KsgA. Mol. Microbiol..

[55] Pletnev P., Guseva E., Zanina A., Evfratov S., Dzama M., Treshin V., Pogorel’skaya A., Osterman I., Golovina A., Rubtsova M. (2020). Comprehensive Functional Analysis of Escherichia Coli Ribosomal RNA Methyltransferases. Front. Genet..

[56] Andersen N.M., Douthwaite S. (2006). YebU Is a M5C Methyltransferase Specific for 16 S RRNA Nucleotide 1407. J. Mol. Biol..

[57] Kimura S., Suzuki T. (2010). Fine-Tuning of the Ribosomal Decoding Center by Conserved Methyl-Modifications in the Escherichia Coli 16S RRNA. Nucleic Acids Res..

[58] Akbergenov R., Duscha S., Fritz A.-K., Juskeviciene R., Oishi N., Schmitt K., Shcherbakov D., Teo Y., Boukari H., Freihofer P. (2018). Mutant MRPS5 Affects Mitoribosomal Accuracy and Confers Stress-Related Behavioral Alterations. EMBO Rep..

[59] Richman T.R., Ermer J.A., Davies S.M.K., Perks K.L., Viola H.M., Shearwood A.-M.J., Hool L.C., Rackham O., Filipovska A. (2015). Mutation in MRPS34 Compromises Protein Synthesis and Causes Mitochondrial Dysfunction. PLoS Genet..

[60] Lagouge M., Mourier A., Lee H.J., Spåhr H., Wai T., Kukat C., Silva Ramos E., Motori E., Busch J.D., Siira S. (2015). SLIRP Regulates the Rate of Mitochondrial Protein Synthesis and Protects LRPPRC from Degradation. PLoS Genet..

[61] Richman T.R., Spåhr H., Ermer J.A., Davies S.M.K., Viola H.M., Bates K.A., Papadimitriou J., Hool L.C., Rodger J., Larsson N.-G. (2016). Loss of the RNA-Binding Protein TACO1 Causes Late-Onset Mitochondrial Dysfunction in Mice. Nat. Commun..

[62] Rudler D.L., Hughes L.A., Perks K.L., Richman T.R., Kuznetsova I., Ermer J.A., Abudulai L.N., Shearwood A.-M.J., Viola H.M., Hool L.C. (2019). Fidelity of Translation Initiation Is Required for Coordinated Respiratory Complex Assembly. Sci. Adv..

[63] Rackham O., Busch J.D., Matic S., Siira S.J., Kuznetsova I., Atanassov I., Ermer J.A., Shearwood A.-M.J., Richman T.R., Stewart J.B. (2016). Hierarchical RNA Processing Is Required for Mitochondrial Ribosome Assembly. Cell Rep..

[64] Mochel F., Slama A., Touati G., Desguerre I., Giurgea I., Rabier D., Brivet M., Rustin P., Saudubray J.-M., DeLonlay P. (2005). Respiratory Chain Defects May Present Only with Hypoglycemia. J. Clin. Endocrinol. Metab..

[65] Gardeitchik T., Mohamed M., Ruzzenente B., Karall D., Guerrero-Castillo S., Dalloyaux D., van den Brand M., van Kraaij S., van Asbeck E., Assouline Z. (2018). Bi-Allelic Mutations in the Mitochondrial Ribosomal Protein MRPS2 Cause Sensorineural Hearing Loss, Hypoglycemia, and Multiple OXPHOS Complex Deficiencies. Am. J. Hum. Genet..

[66] Averina O.A., Vysokikh M.Y., Permyakov O.A., Sergiev P.V. (2020). Simple Recommendations for Improving Efficiency in Generating Genome-Edited Mice. Acta Nat..

[67] Rogers D.C., Peters J., Martin J.E., Ball S., Nicholson S.J., Witherden A.S., Hafezparast M., Latcham J., Robinson T.L., Quilter C.A. (2001). SHIRPA, a Protocol for Behavioral Assessment: Validation for Longitudinal Study of Neurological Dysfunction in Mice. Neurosci. Lett..

[68] Huang C.-C., Hsu M.-C., Huang W.-C., Yang H.-R., Hou C.-C. (2012). Triterpenoid-Rich Extract from *Antrodia Camphorata* Improves Physical Fatigue and Exercise Performance in Mice. Evid.-Based Complement. Altern. Med..

[69] Wang S.-Y., Huang W.-C., Liu C.-C., Wang M.-F., Ho C.-S., Huang W.-P., Hou C.-C., Chuang H.-L., Huang C.-C. (2012). Pumpkin (Cucurbita Moschata) Fruit Extract Improves Physical Fatigue and Exercise Performance in Mice. Molecules.

[70] Chen K., Li N., Fan F., Geng Z., Zhao K., Wang J., Zhang Y., Tang C., Wang X., Meng X. (2021). Tibetan Medicine Duoxuekang Capsule Ameliorates High-Altitude Polycythemia Accompanied by Brain Injury. Front. Pharmacol..

[71] Li Q., Wang Y., Cai G., Kong F., Wang X., Liu Y., Yang C., Wang D., Teng L. (2015). Antifatigue Activity of Liquid Cultured *Tricholoma Matsutake* Mycelium Partially via Regulation of Antioxidant Pathway in Mouse. BioMed Res. Int..

[72] Kaur H., Kaur R., Jaggi A.S., Bali A. (2020). Beneficial Role of Central Anticholinergic Agent in Preventing the Development of Symptoms in Mouse Model of Post-Traumatic Stress Disorder. J. Basic Clin. Physiol. Pharmacol..

[73] Hult E.M., Bingaman M.J., Swoap S.J. (2019). A Robust Diving Response in the Laboratory Mouse. J. Comp. Physiol. B.

[74] Reger M.L., Hovda D.A., Giza C.C. (2009). Ontogeny of Rat Recognition Memory Measured by the Novel Object Recognition Task. Dev. Psychobiol..

[75] Gaskin S., Tardif M., Cole E., Piterkin P., Kayello L., Mumby D.G. (2010). Object Familiarization and Novel-Object Preference in Rats. Behav. Process..

[76] Antunes M., Biala G. (2012). The Novel Object Recognition Memory: Neurobiology, Test Procedure, and Its Modifications. Cogn. Process.

[77] Eagle A., Wang H., Robison A. (2016). Sensitive Assessment of Hippocampal Learning Using Temporally Dissociated Passive Avoidance Task. BIO-PROTOCOL.

[78] Xiang C., Li Z.-N., Huang T.-Z., Li J.-H., Yang L., Wei J.-K. (2019). Threshold for Maximal Electroshock Seizures (MEST) at Three Developmental Stages in Young Mice. Zool Res..

[79] Ågamo A., Ögren S.O., Abizaid A., Aboitiz F., Absalom A., Adell A., Adelt I., Alici Y., Allgulander C., Almeida O. (2011). Encyclopedia of Psychopharmacology: A Springer Live Reference.

[80] Graham B. (2002). Noninvasive, in Vivo Approaches to Evaluating Behavior and Exercise Physiology in Mouse Models of Mitochondrial Disease. Methods.

[81] Serchov T., van Calker D., Biber K. (2016). Light/Dark Transition Test to Assess Anxiety-like Behavior in Mice. BIO-PROTOCOL.

[82] Bourin M., Hascoët M. (2003). The Mouse Light/Dark Box Test. Eur. J. Pharmacol..

[83] Lojda Z., Gossrau R., Schiebler T.H. (1979). Enzyme Histochemistry: A Laboratory Manual.

[84] Luna L. (1993). Histopathologic Methods and Color Atlas of Special Stains and Tissue Artifacts.

[85] Debacq-Chainiaux F., Erusalimsky J.D., Campisi J., Toussaint O. (2009). Protocols to Detect Senescence-Associated Beta-Galactosidase (SA-Βgal) Activity, a Biomarker of Senescent Cells in Culture and in Vivo. Nat. Protoc..

[86] Suvarna S.K., Layton C., Bancroft J.D. (2019). Bancroft’s Theory and Practice of Histological Techniques.

[87] Aibara S., Andréll J., Singh V., Amunts A. (2018). Rapid Isolation of the Mitoribosome from HEK Cells. J. Vis. Exp..

[88] Dayal A.A., Medvedeva N.V., Nekrasova T.M., Duhalin S.D., Surin A.K., Minin A.A. (2020). Desmin Interacts Directly with Mitochondria. Int. J. Mol. Sci..

[89] Reschke M., Clohessy J.G., Seitzer N., Goldstein D.P., Breitkopf S.B., Schmolze D.B., Ala U., Asara J.M., Beck A.H., Pandolfi P.P. (2013). Characterization and Analysis of the Composition and Dynamics of the Mammalian Riboproteome. Cell Rep..

[90] Golde W.T., Gollobin P., Rodriguez L.L. (2005). A Rapid, Simple, and Humane Method for Submandibular Bleeding of Mice Using a Lancet. Lab. Anim..

[91] Diehl K.H., Hull R., Morton D., Pfister R., Rabemampianina Y., Smith D., Vidal J.M., van de Vorstenbosch C. (2001). European Federation of Pharmaceutical Industries Association and European Centre for the Validation of Alternative Methods A Good Practice Guide to the Administration of Substances and Removal of Blood, Including Routes and Volumes. J. Appl. Toxicol..

