# Supplementary material for: Mitochondrial rRNA Methylation by Mettl15 Contributes to the Exercise and Learning Capability in Mice
Source: Int J Mol Sci. 2022 May 27;23(11):6056. doi: 10.3390/ijms23116056 (PMC9181494; doi:10.3390/ijms23116056)
Supplement: Supplementary file 1 [file ijms-23-06056-s001.zip › ijms-1689095-supplementary.pdf]

International Journal of *Molecular Sciences*

MDPI

Supplementary materials

Mitochondrial rRNA methylation by Mettl15 contributes to the exercise and learning capability in mice

Olga A. Averina[1,2†], Ivan G. Laptev[2†], Mariia A. Shepeliuk[3], Oleg A. Permyakov[1], Sofya S. Mariasina[1], Alyona I. Nikiforova[4], Vasily N. Manskikh[2], Olga O. Grigorieva[1], Anastasia K. Bolikhova[5], Gennady A. Kalabin[6], Olga A. Dontsova[2,3,7,8], Petr V. Sergiev[1,2,3,7*]

[1] Institute of functional genomics, Lomonosov Moscow State University, Moscow, Russia; averina.olga.msu@gmail.com (O.A.A.); norad_m@mail.ru (O.A.P.); sm1024sm@yandex.ru (S.S.M.); grig_forever@mail.ru (O.O.G.);
[2] Belozersky Institute of Physico-Chemical Biology, Lomonosov Moscow State University, Moscow, Russia; whiteswan92@gmail.com (I.G.L.); manskikh@mail.ru (V.N.M.);
[3] Center of life sciences, Skolkovo Institute of Science and Technology, Moscow, Russia; mshep98@mail.ru (M.A.S.);
[4] Institute of Mitoengineering, Lomonosov Moscow State University, Moscow, Russia; rabio@mail.ru (A.I.N.);
[5] Faculty of Bioengineering and Bioinformatics, Lomonosov Moscow State University, Moscow, Russia; anastasia_b7@mail.ru (A.K.B.);
[6] Peoples' Friendship University of Russia (RUDN), Moscow, Russia; kalabin-ga@rudn.ru (G.A.K.);
[7] Department of chemistry, Lomonosov Moscow State University, Moscow, Russia; olga.a.dontsova@gmail.com (O.A.D.);
[8] Shemyakin-Ovchinnikov Institute of Bioorganic Chemistry, Moscow, Russia
*Correspondence: petya@genebee.msu.ru;
†These authors have contributed equally to this work and share first authorship

1. Supplementary Materials and Methods

1.1. Mettl15 gene inactivation

For inactivation of *M. musculus Mettl15* gene in mouse zygotes we used the same sgRNA sequence (GCATACTGAATCTAAAGCTG) as was previously used for *Mettl15* gene inactivation in murine cell lines [1]. To prepare sgRNA we amplified a fragment of pX458 plasmid [2] coding the constant part of sgRNA appending it with T7 promoter and the guide sequence shown above. The amplicon was used for *in vitro* transcription with MEGAscript kit (Thermo scientific). Purified sgRNA was mixed with mRNA coding for *S. pyogenesis* Cas9 (Thermo scientific) to a final concentration 12 ng/uL sgRNA and 25 ng/uL Cas9 mRNA and used for murine zygotes microinjection.

The genome-edited mice were generated as previously described [3]. All manipulations were conducted in compliance with the protocol №182 approved by the Local Bioethics Commission of the Research Center "Institute of Mitoengineering of Moscow State University" LLC, (Moscow, Russia) (http://www.vec-msu.ru/). The mice were kept in conditions free from pathogenic microorganisms, in individually ventilated cages (IVC system, TECNIPLAST S.p.A., Italy), with free access to granulated autoclaved chow and reverse osmosis water; with light mode 12/12 (light on at 09:00); in rooms with an air exchange rate of at least 15 rev / h, with an air temperature of 20-24°C, humidity 30-70%. The standard conditions of keeping in a barrier vivarium made it possible to stabilize the homeostasis and behavior of the animals.

1.2. Analysis of RNA modification

For the monitoring of the modification status of the 12S rRNA nucleotide C839 total RNA was isolated from the hearts, liver and kidneys of four *Mettl15*$^{-/-}$ mice and four mice of the control group by Trizol extraction. 12S rRNA fragment was isolated by a previously published procedure [1] using 5′-biotinylated oligodeoxyribonucleotide (5′-[biotin]- TGTTAAGTTTAATTTAATTTGAGGAGGGTGACGGGCGGTG) complemen-

tary to the region interest of the 12S rRNA. Total RNA (2 mg/mL, 6 mg) and the oligonucleotide (200 pmol/mL) were heated in 6x SSC buffer for 5 min at 95 °C and left in thermostat to anneal. After cooling to ~37 °C, RNase T1 (Thermo Scientific) was added to the mixture to the final concentration of 1 unit/ul and incubated for 1 h at 37 °C. Then the solution was mixed with Streptavidin Sepharose High performance (GE Healthcare) and incubated 20 min at room temperature with rotation. The resin was washed 3 times with 3x SSC, 4 times with 1x SSC and 4 times with 0.1x SSC. 12S rRNA fragment was eluted from the resin by adding elution buffer (0.1x SSC, 6M urea) and incubation for 5 min at 75 °C. RNA from the eluate was precipitated with isopropanol overnight at -20 °C. Eluted RNA fragment was separated via 12% denaturing polyacrylamide gel electrophoresis and digested with 1 unit/ul RNase T1 in 50 mM ammonium citrate for 3 hours at 37 °C. Digestion mixture (0.5 ul) was mixed with 50 mg/mL 2,5-dihydroxybenzoic acid in 0.5% trifluoroacetic acid and 30% acetonitrile (1.5 ul) and was left to air dry at room temperature. MALDI mass spectrometry was performed in a reflector mode on an Ultraflex III BRUKER equipped with a UV laser (Nd, 335 nm) detecting positive ions.

1.3. Histopathological examination

During the necropsy all changes were recorded. The brain, heart, spleen, kidneys, liver, testis, lungs, tibialis anterior and soleus muscles were examined histologically. The specimens were fixed with 10% buffered formalin solution (pH 7.4), dehydrated with absolute isopropanol, and paraffin-embedded. Microtome sections with thickness of 3 μm were stained with hematoxylin and eosin. All examinations were done by professional laboratory animal pathologist and images were recorded using an Axiosope A1 microscope with a CCD-camera AxioCam MRc.5 (Carl Zeiss).

To stain SA-beta-galactosidase-positive senescent cells we used the protocol for paraffin sections created by V.N. Manskikh: organ samples (liver, kidney, spleen, heart, brain, skeletal muscles, skin, small intestine) were fixed with Baker's formol-calcium solution (1g $CaCl_2$, 10 ml 37% formaldehyde, 90 ml water) [4] during 24 h at +4°C, washed in distillated water for 24 h, dehydrated in 3 changes of absolute acetone at +4°C for 2 h in each, cleared 1 h in benzene and embedded in paraffin with Z. Lojda et al. method for enzyme histochemistry [5]. Microtome sections with thickness of 5 μm were stained 3 h with X-gal solution (20 mg X-gal, 1 ml Dimethylformamide, 5 ml 1,65% $K_3[Fe(CN)_6]$, 5 ml 2,11% $K_4[Fe(CN)_6]$, 70 ml 0,1 M citrate buffer pH=6.0) at suboptimal pH=6.0 and 37°C as recommended [6] and counterstained with nuclear red. Number of senescent cells in full organ section was counted manually and normalized to 1 mm^2 of section area with ImageJ software on organ photomicrographs.

To stain the mitochondria, the kidney and liver samples were fixed 48h at +4°C with Regaud fixative (mixture of 37% formaldehyde, 1 part, and 3% potassium dichromate, 3 parts), incubated in 3% potassium dichromate solution for 72 h, washed in distilled water for 24 h, dehydrated with 99,7% isopropanol, embedded in paraffin and sectioned with microtome. Sections (2 μm) were stained with saturated aniline acid fuchsin 30 min at 60°C and differentiated in ethanol solutions of picric acid according to classical Altmann's method [7]. Area of mitochondria was estimated with ImageJ software on 3 photomicrographs (100x) of sections of renal tubules in kidney cortex.

For study of myopathy lesions, fresh samples of skeletal muscles were sectioned with cryotome. Unfixed cryotome sections with thickness of 5 μm were stained with Gomori trichrome [8] for elucidation of myofibers with signs of mitochondrial abnormalities. Sections were stained with filtered hematoxylin for 5 minutes, washed with distilled water, stained with filtered Gomori trichrome stain (Fast Green FCF 0.3 g, Chromotrope 2R 1.2 g, Phosphotungstic acid 0.6 g, Glacial acetic acid 1 ml, Distilled water 100 ml, pH 3.4) for 15 minutes, washed with distilled water and rinse briefly in 0.5% acetic acid for differentiation. Stained sections were dehydrated with 95% ethanol, cleared with xylene and mounted.

Glycogen was estimated in the PAS-stained histologic section [4,8]. Samples of gastrocnemius muscle and liver were fixed with glycogen-preserved Schabadasch fixative ($Cu(NO_3)_2$ - 1,8 g, $Ca(NO_3)_2$ – 0.9 g, 37% formaldehyde – 10 ml, 96% ethanol – 100 ml) for 24 h and paraffin-embedded. Paraffin sections (3 µm) were stained with standard PAS-procedure without counterstain. For PAS staining, sections were oxidized with 0.5% periodic acid for 5 min, rinsed in distilled water, stained with Schiff reagent for 15 min and rinsed in running tap water for 5 min. Photomicrographs were taken with microscope AxioScope A1 and camera MRc.5 (Carl Zeiss, Germany). Area of PAS-positive glycogen deposits was estimated with Image J software.

For mitochondria staining formalin fixed paraffin embedded slices with mice organs were preheated at 56°C for 5 min and submerged into xylol. After 3 incubations in xylol and 2 incubations in ethanol for 5 min each, the slides were rehydrated in TBS for 15 min. Epitope was retrieved in preheated to 92°C citrate buffer (10 mM sodium citrate pH 6.0, 05% Tween-20) for 12 min in a boiling water bath with the following cooling at room temperature. After that, the slides were blocked with 2% BSA in TBS at room temperature at the shaker for 1 hour. Then slides were washed by TBS and incubated with antibodies against VDAC1 (Abcam ab15895, 1:180) in 2% BSA in TBST and were put into the humidified chamber at +4°C overnight. The slides were washed 5 times for 5 min by 2% BSA in TBST. After that, the slides were incubated with the Cy5-labeled goat anti-rabbit antibodies in 2% BSA in TBST (Invitrogen, A10523, 1:200) for 1 hour at room temperature in the humidified chamber with the following 5 washes by 2% BSA in TBST for 5 min each. Then the slides were mounted in mowiol with DAPI and let to solidify overnight in the dark.

The images were captured on Nikon C2 microscope using the same parameters for each image. For comparison, the mean ROI intensity of square micrometer for each captured image was used. For statistical analysis unpaired t-test was used. For each mouse line, samples of 5 mice were analyzed with 5 images of different fields of view of each slide.

1.4. Experimental groups

All experiments were performed on no less than 8 animals in each group except for b-galactosidase activity staining of 1 year old wild type mice (n=2) and *Mettl15*$^{-/-}$ knockout mice (n=2).

1.5. Statistical analysis

Statistical data analysis was performed using the nonparametric Mann–Whitney U test. The chosen significance level was $p < 0.05$. The results are presented as median and 0.25–0.75 quartile range.

1.6. Body mass and temperature

Body mass was measured by a laboratory balance. Temperature was measured by electronic thermometer inserted into the rectum.

1.7. Grip force test

The test is designed to evaluate the maximum voluntary strength (grip force) and is a common method of mice and rats limb strength and neurological deficit determination [9]. The lever of the device acts as a mouse support, the animal placed parallelly to the table surface in horizontal position and with abrupt hand movement mouse is being pulled by the tail until it lets the lever go. This procedure is repeated 10 times with 15-30 s range until attempts; five maximum values are averaged.

1.8. Forced swimming test

The test is designed to evaluate the physical endurance of laboratory animals [10,11]. The analyzed value is the animal maximum swimming time representing physical performance capability. A mouse with 13% of mouse body weight load fixed on the middle part of the tail was placed in a cylinder (10 cm diameter, 30 cm height) filled

with water (22-24°C) to the 20 cm height. The animal's refusal to swim expressing in the complete submerging under the water surface for 5 seconds period is considered as the end of the test. The parameters of the test were set to minimize the harm to an animal while avoiding erroneous underestimation of the swimming time. The weight of a load (13%) was set on the basis of our previous experience and data from the literature. In the analogous studies the load used ranged from 4 % [12] to 15% [13] of a body weight. The time of complete submerging under the water surface (5 s) prior to a rescue was set to avoid an underwater swim trauma which may be caused by >30 s submerging [14] and discrimination against voluntary underwater swim which may last for 4–6 seconds [15].

1.9. Novel Object Recognition (NOR) test

The test is designed to evaluate general exploratory activity and object recognition memory in rodents [16,17]. Basically, in the NOR task, there are no positive or negative reinforcers, and this methodology assesses the natural preference for novel objects displayed by rodents [18]. In the "Open Field" installation at a distance of 10 cm from the side, opposite each other in opposite quadrants, two objects of the same shape and color and a mouse are placed for 5 minutes. Next day the test is repeated, one of the objects was replaced with a new one, different by color and shape from the initial one. Both on the first and on the second experimental days, the interest of mice in objects is recorded - the number of approximations, sniffing, and direct contacts. The results on the interest of mice in objects on the first day can be considered as their general exploratory activity. On the second experimental day, normally, when an object is replaced, animals tend to explore it; when memory is impaired, mice do not perceive the replaced object as a quality, which is reflected in a decrease in exploratory behavior in this object relative to the control group [18].

1.10. Passive avoidance test

This fear-motivated test is designed to evaluate memory in rodents. The test unit consists of a dark chamber with an electrode floor and light chamber, each 19x38 cm in size, separated by a wall with a sliding door. The mouse is placed in a brightly lit compartment for 60 seconds for acclimatization, after which the door is opened and the latency period of passage to the compartment with the electrode floor is recorded. The transition is considered to be the movement of the animal of all 4 paws and tail into the dark compartment, then the door is closed and after 2 seconds an electric shock is applied across the floor, after which the mouse is left for 30 seconds in the dark compartment. One day later, the animal is again placed in the light compartment where no shock was delivered, and the latency to cross through the gate between the compartments is assessed with the door open [19]. The mice are stimulated with an alternating voltage of 40 V, a frequency of 50 Hz, with an average integral current limited to 3.9 mA. It is known that these parameters (40 V with a current limitation of 4 mA) do not lead to the characteristic behavior of induced electroshock seizures in adult mice [20], but cause a moderate vocalization and a visual increase in motor activity, which is sufficient for the formation of a memory footprint the day after exposure [21]. Memory performance is positively correlated with the latency of passage to the dark compartment on the second experimental day, the better the memory, the greater the latency.

1.11. Hot plate test

This test is designed to assess nociception (response to painful stimuli). The mouse is placed on a hot plate at a controlled temperature (52-55.0°C) and the waiting time until the first reaction of the hind paw is recorded; the response of the hind paw is considered to be licking it [22].

1.12. The light/dark transition test (LDT)

The test is one of the most widely used tests to measure anxiety-like behavior in mice [23]. The LDT test is based on the innate aversion of rodents to brightly illuminated areas and on the spontaneous exploratory behavior of rodents in response to mild

stressors, that is, novel environment and light [24]. The test unit consists of a dark and light chambers, each 19x38 cm in size. The latency of passage from bright to the dark chamber was the studied research parameter.

1.13. The box-maze test

Likewise LDT test, the box-maze test [25] is based on rodents avoidance of brightly illuminated areas. The box-maze apparatus contains illuminated central box with five holes leading to an immediate dead end that are 2.5 cm depth and the escape hole that is connected to the standard housing cage. Mice were placed into the illuminated box-maze and allowed to explore it and behave freely for up to 2 min. The latency time to fully enter the escape hole is recorded. Mice are subjected to a total of four such trials, all on the same day. Over the course of the four trials, mice learn the location of the escape hole and exit the maze through it with progressively shorter escape latency. After 48 hours the procedure is repeated and mice are subjected to the only one trial.

1.14. Sucrose gradient profiling of mitochondrial ribosomes

Mitochondria and mitochondria ribosomes were isolated combining methods described in [26,27] with some changes. Mice were sacrificed by cervical dislocation and liver was washed with ice cold PBS, dried with paper cloth and homogenized in teflon Dounce homogenizer in MIBSM buffer (50 mM HEPES-KOH, pH 7.5, 70 mM sucrose, 210 mM mannitol, 10 mM KCl, 1.5 mM $MgCl_2$, 2 mM EDTA, 1mM DTT, protease inhibitors). Lysate was centrifuged at 700×g 15 min at +4°C and supernatant was collected and centrifuged at 7 000×g 20 min at +4°C. The mitochondria pellet was homogenized in MIBSM buffer and centrifuged at 7 000×g 20 min at +4°C. The pellet after centrifugation was resuspended in 1 ml of SEM (20 mM HEPES-KOH, 250 mM sucrose, pH 7.5, 1 mM EDTA) and loaded on top of the step sucrose gradient (2 mL 60%, 4 mL 32%, 2 mL 23% and 2 mL 15% sucrose in 20 mM HEPES-KOH, pH 7.5, 1 mM EDTA) and centrifuged in SW41Ti rotor at 27 000 rpm for 1 h at +4°C. Mitochondria (brown migrating band between 60% and 32% sucrose) were collected from tubes, resuspended in equal volume of gradient buffer without sucrose (20 mM HEPES-KOH, pH 7.5, 1 mM EDTA) and centrifuged at 8 000×g 10 min at +4°C. Pellet of mitochondria were snap-freezed in liquid nitrogen and stored at -80°C.

Mitoribosome profiles were obtained as described [1]. Mitochondria were defrosted in ice and lysed in two volumes of lysis buffer (25 mM HEPES-KOH, pH 7.5, 100 mM KCl, 20 mM $Mg(OAc)_2$, 2% Triton X-100, 2 mM DTT, protease inhibitors) for 10 min on ice. Lysates were cleared by centrifugation at 16 000×g for 30 min at +4°C, were loaded on top of 0.4 mL sucrose cushion (20% sucrose, 25 mM HEPES-KOH, pH 7.5, 50 mM KCl, 10 mM $Mg(OAc)_2$, 0.5% Triton X-100, 2 mM DTT) and centrifuged in MLA-130 rotor at 75 000 rpm for 2 h at +4°C. Pellet was resuspended in resuspension buffer (25 mM HEPES-KOH, pH 7.5, 50 mM KCl, 20 mM $Mg(OAc)_2$, 0.05% n-Dodecyl β-D-maltoside, 2 mM DTT) and loaded on top of 10 mL of 10%-30% sucrose gradient (25 mM HEPES-KOH, pH 7.5, 50 mM KCl, 10 mM $Mg(OAc)_2$, 2 mM DTT) and centrifuged in SW41Ti rotor at 24 000 rpm for 16 h.

Mitoribosome gradients were fractionated into 0.5 mL fractions using ACTA purifier (GE Healthcare) while monitoring absorbance at 254 nm. From 14 fractions from each gradient proteins were isolated by TCA/Deoxycholate- precipitation as described in [28]. Pellets were dissolved in equal volume of 1x Laemmli buffer and equal volume of each fraction was loaded on SDS page for western blot analysis.

1.15. Western blot analysis

For western blot analysis RIPA lysates of mice organs were used. Organs were put in liquid nitrogen, defrosted in ice in RIPA buffer with Halt Protease and Phosphatase Inhibitor Cocktail (Thermo Scientific), homogenized 2 times in Precellys Evolution homogenizer (Bertin Technologies) at 6 800 rpm for 30 sec. Lysates were cleared for 30 min

at 16 000×g at +4°C. Protein concentration was measured by Bradford assay and 30 μg of each lysate were used for western blotting.

For detection of the proteins the following antibodies were used: RBFA antibody (PA5-59587, Invitrogen), MRPS34 antibody (PA5-59872, Invitrogen), MRPL48 antibody (ab194826, Abcam), AKT (9272, Cell Signaling Technology), pAKT (Thr308) (4056, Cell Signaling Technology), LC3A/B (PA1-16931, ThermoFisher Scientific), SDHB (PA5-29843, ThermoFisher Scientific), aTubulin (T6199, Sigma).

1.16. Quantitative RT–PCR

Total RNA was extracted from mice tissue homogenates using QIAzol Lysis Reagent (Qiagen). cDNA was synthesized from total RNA using Maxima First Strand cDNA Synthesis Kit for RT-qPCR (Thermo Scientific™). The RT-PCR was performed on individual cDNAs by using SYBR® Green PCR master mix in the CFX384 Touch Real-Time PCR System. The primer sequences are available in the supplementary materials. The mRNA expression was calculated by the 2−ΔΔCT method and normalized to the expression of *Gapdh*.

Copy number of mtDNA was estimated by quantitative PCR as previously described [1]. Quantity of mtDNA was analyzed using the primers for the 12S rDNA, while nuclear DNA copy number was analyzed using the primers for KIAA1456 gene (5′-CTTCCTTCTCTTACCTGCACGCC, 5′-GGTTACCAATGTCAGCGACGAGG).

1.17. Metabolome analysis

For the analysis of lactate and glucose concentration, blood samples were taken from the facial vein [29] and analyzed by a portable biochemical analyzer Accutrend Plus (Roche Diagnostics, Germany). For complete NMR analysis of metabolome, blood was collected by cardiac puncture [30]. The procedure was performed on anesthetized animals (inhalational anesthesia, isoflurane) without postanesthetic recovery. After collection in CAT Serum Sep Clot activator tubes, the blood was centrifuged for 20 min at 2000×*g* to separate the plasma from cells. Then 200 uL of plasma was aliquoted and used immediately for metabolite extraction by 400 uL of 1:1 mixture of cold HPLC-grade methanol and chloroform. After intensive vortexing and incubation during 10 min at 5°C, tubes were centrifuged for 30 min at 14000 g at 5°C. Water (upper) layer was moved to a new tube and dried using vacuum concentrator SpeedVac at room temperature during 3 h up to complete solvent evaporation. Then samples were freeze-dried during 3 h.

Before NMR measurements, the solid metabolites were dissolved in 550 uL of buffer (50 mM sodium phosphate at pH 7.0, 0.107 mM d_6-DSS, 0.13 mg/mL NaN_3 dissolved in D_2O) and loaded into a standard 5 mm NMR tube.

All NMR spectra were acquired on a Bruker Avance 700 MHz NMR spectrometer equipped with a Z-axis-gradient 5 mm HCN Prodigy cryoprobe using automatic sampler with storage temperature of 5°C. Spectra measurements were performed at 35°C.

One-dimensional ^1H NMR spectra were acquired from each sample using the standard 1D NOESY-presat experiment. A total of 400 scans were accumulated for plasma samples. $^1H,^{13}C$-HSQC spectra were measured for one sample from each group to confirm the metabolites assignments.

All steps of 1D spectra processing and metabolite identification were performed using Chenomx NMR Suite program. $^1H,^{13}C$-HSQC spectra were analyzed in TopSpin program.

2. Supplementary Tables

Supplementary Table S1. Primer list for RT qPCR analysis of gene expression

RNA	Forward primer	Reverse primer
12S rRNA	CTCAAAGGACTTGGCGGTAC	GTTTGCTGAAGATGGCGGTA
16S rRNA	TGAAATTTCGGTTGGGGTGA	TCCCTAGGGTAACTTGGTCC
18S rRNA	GTAACCCGTTGAACCCCATT	GGCCTCACTAAACCATCCAAT
Gapdh	TGCACCACCAACTGCTTAGC	GGCATGGACTGTGGTCATGAG
mt-Nd1	TCCCCTACCAATACCACACC	CGGCTCGTAAAGCTCCGAAT
mt-Nd2	AGGGGCATGAGGAGGACTTA	TGAGTAGAGTGAGGGATGGGT
mt-Nd3	ACAAGCTCTGCACGTCTACC	TGCTCATGGTAGTGGAAGTAGAA
mt-Nd4L	CCACATTACTATGCCTGGAAGG	TGGTGATGGGGATTGGTATGG
mt-Nd4	ACCCGATGAGGGAACCAAAC	AGCGTCTAAGGTGTGTGTTGT
mt-Nd5	TGACCCAGACCTCATAAACCC	GACTGGAATGCTGGTTGGTG
mt-Nd6	CCCGCAAACAAAGATCACCC	TCTTGATGGTTTGGGAGATTGGT
mt-Cytb	ACCTCAAAGCAACGAAGCCT	TGGGTGTTCTACTGGTTGGC
mt-Co1	TCAATGGGAGCAGTGTTTGC	GATGGCGAAGTGGGCTTTTG
mt-Co2	GACGAAATCAACAACCCCGT	TAGCAGTCGTAGTTCACCAGG
mt-Co3	CTGCTGACCTCCAACAGGAA	GGGCTTGATTTATGTGGTTTCGT
mt-ATP6	TTGCCCACTTCCTTCCACAA	GCTGTAAGCCGGACTGCTAAT
mt-ATP8	AGTCTCATCACAAACATTCCCA C	AGGGGTTTTTACTTTTATGGTTGT T
Ndufv1	GATGTGTTTGTGGTGCGTGG	GAATTGCGTTCTCGGCCAAA
Ndufv3	GTACCTAAGTGCGACCTCCG	GACAGCCTCACTACAGAAACCA
Ndufa3	TCAACAAGGCCACACCCTAC	ACAGGTTCTTCAGCCAGTCC
Ndufb6	AGGAGACGATGGCTGAAGGA	AACAGCGAAGAGACTGGAGC
Ndufs7	CGGGCTGAGTATGTGGTGAC	CATCTTGTTGGTAAGCGTGC
Ndufs8	GAGCCTGCCACCATCAACTA	TGTCATAGCGTGTCGTTCGG
Ndufb9	AGAATCAGCATCCTCAGCCG	CAGCTCTCCATCCTCAGCTTCTT
Ndufb11	CCTCCAGGGCTGTAATCGC	ACGAAGGTGGTCCCAAAGAC
Atf4	AGACACCGGCAAGGAGGAT	CATCCAACGTGGTCAAGAGC
Tfam	GCATACAAAGAAGCTGTGAGC AA	GCTGAACGAGGTCTTTTTGGT
Fgf21	AAATCCTGGGTGTCAAAGCCT	TTGTAACCGTCCTCCAGCAG

3. Supplementary Figures

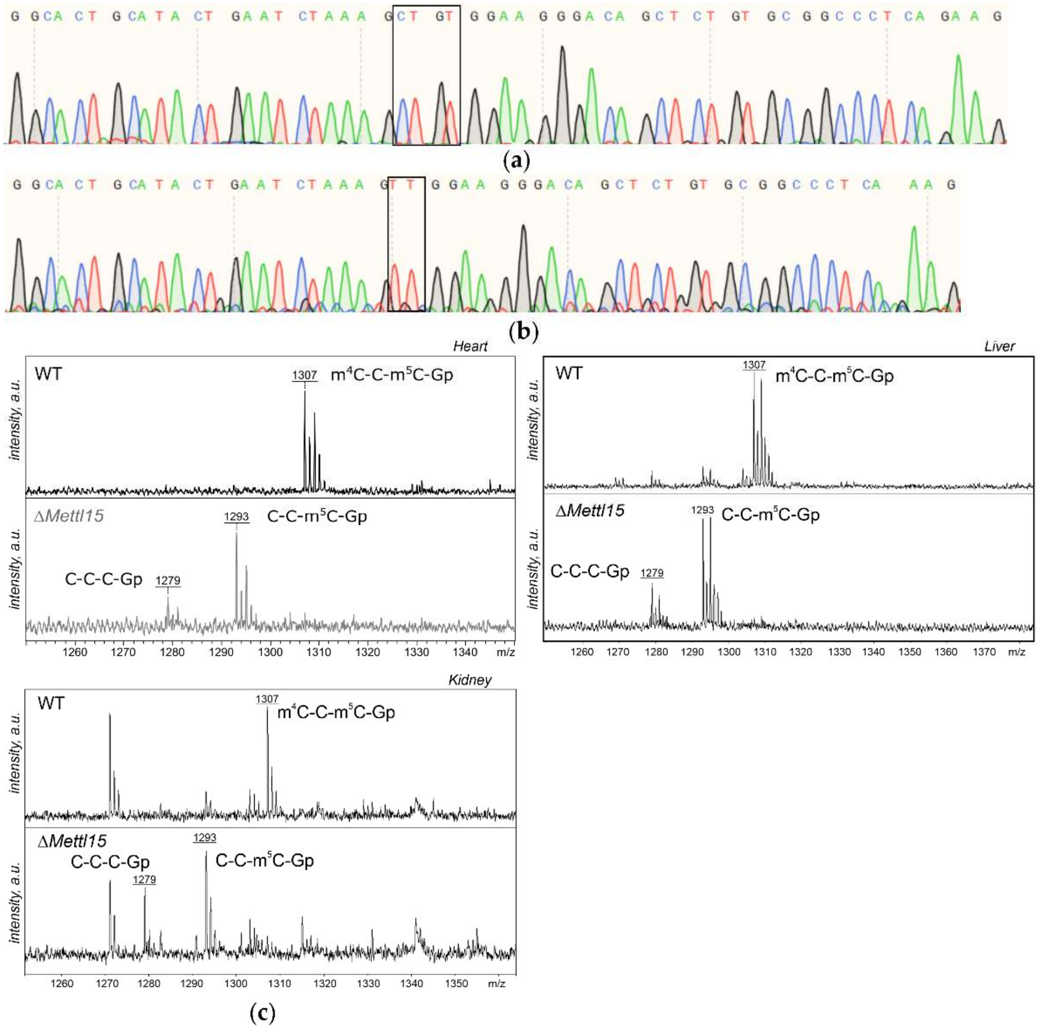

Supplementary Figure S1. Inactivation of *Mettl15* gene. (**a**) Sequence of *Mettl15* gene part corresponding to the site of CRISPR/Cas9 cleavage in the wild type mice. (**b**) Sequence of *Mettl15* gene part in the *Mettl15*$^{-/-}$ knockout mice. (**c**) Mass spectra of the 12S rRNA fragment with C839 (human numbering) nucleotide extracted from heart, liver and kidney (organs are designated above the panels). Upper panels correspond to the wild type mice, while the lower panel corresponds to the *Mettl15*$^{-/-}$ mice. Mass of 1307 Da corresponds to fully methylated fragment, 1293 Da – fragment with one methyl group, 1279 Da – fragment without methyl groups.

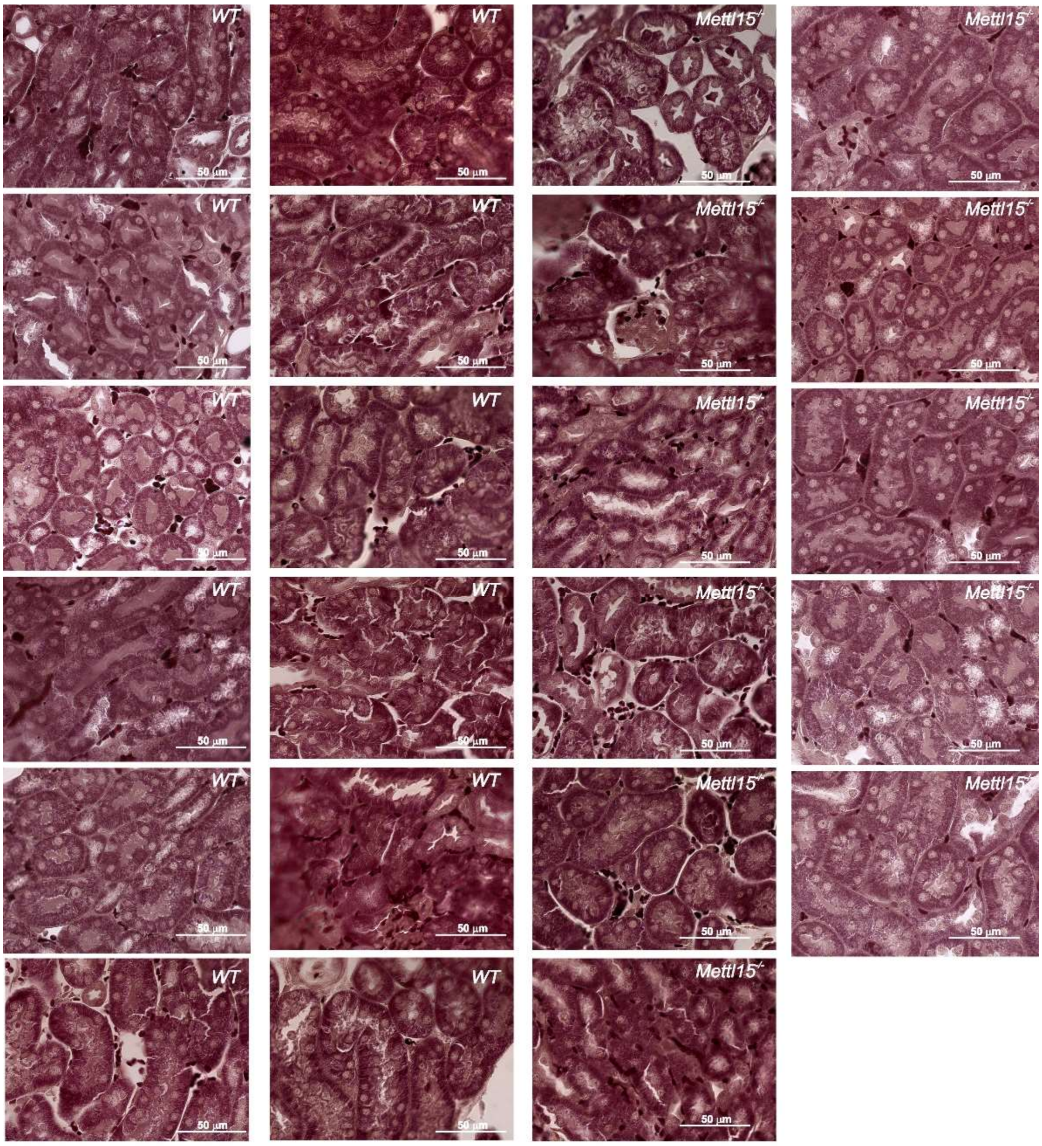

(**a**)

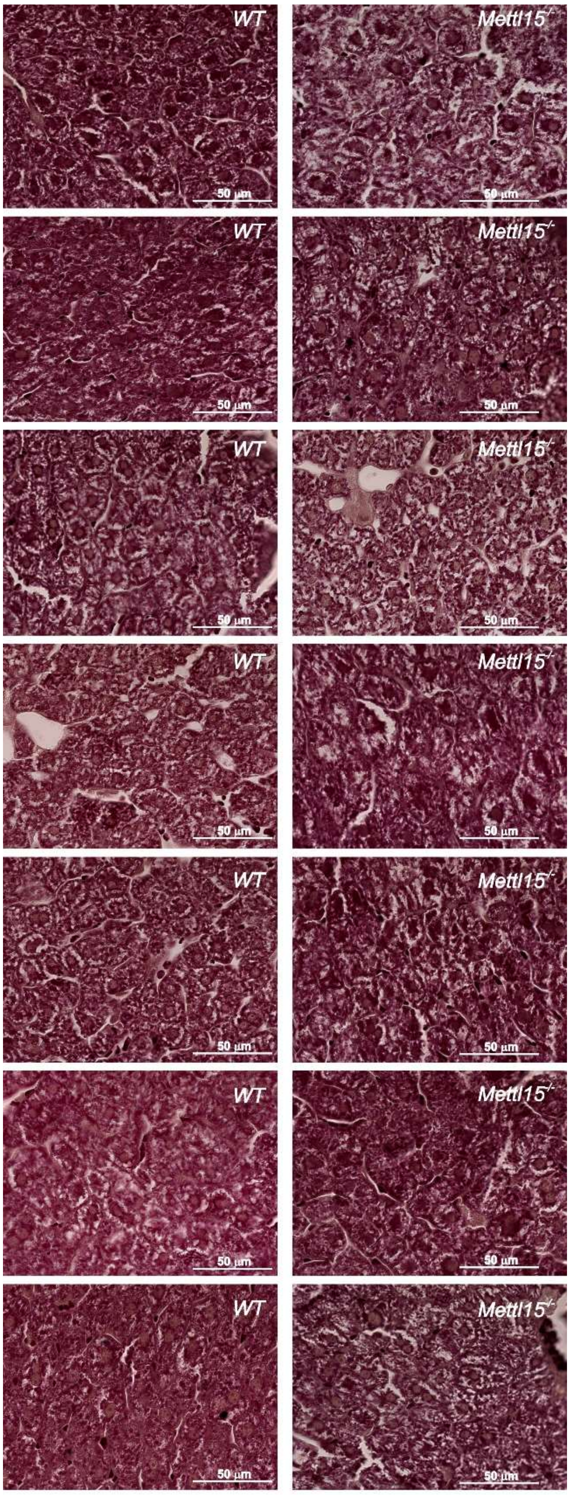

(b)

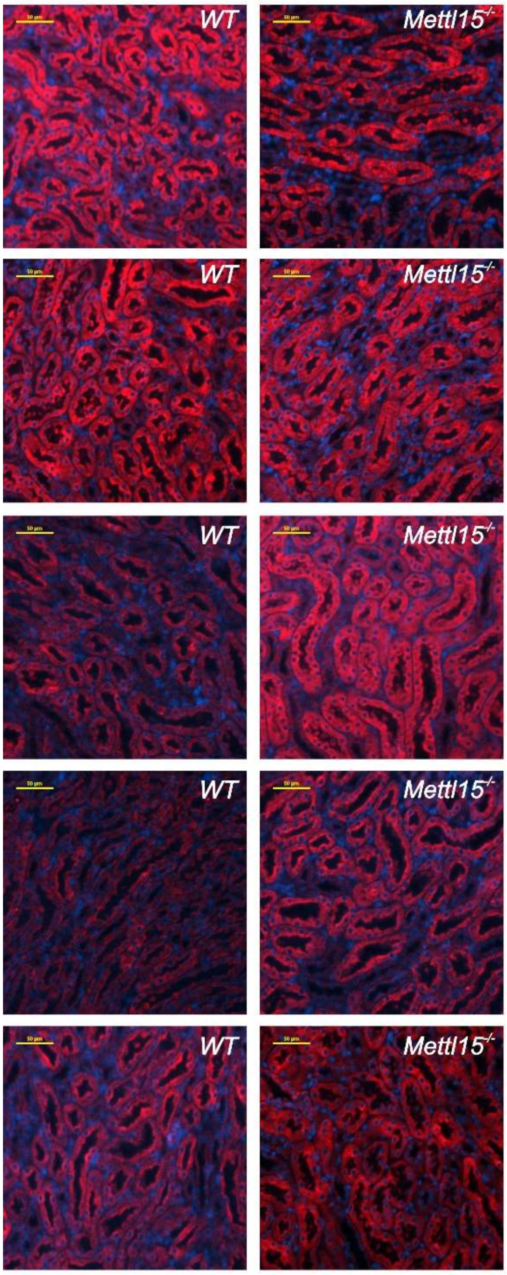
WT
Mettl15-/-
WT
Mettl15-/-
WT
Mettl15-/-
WT
Mettl15-/-
WT
Mettl15-/-

(c)

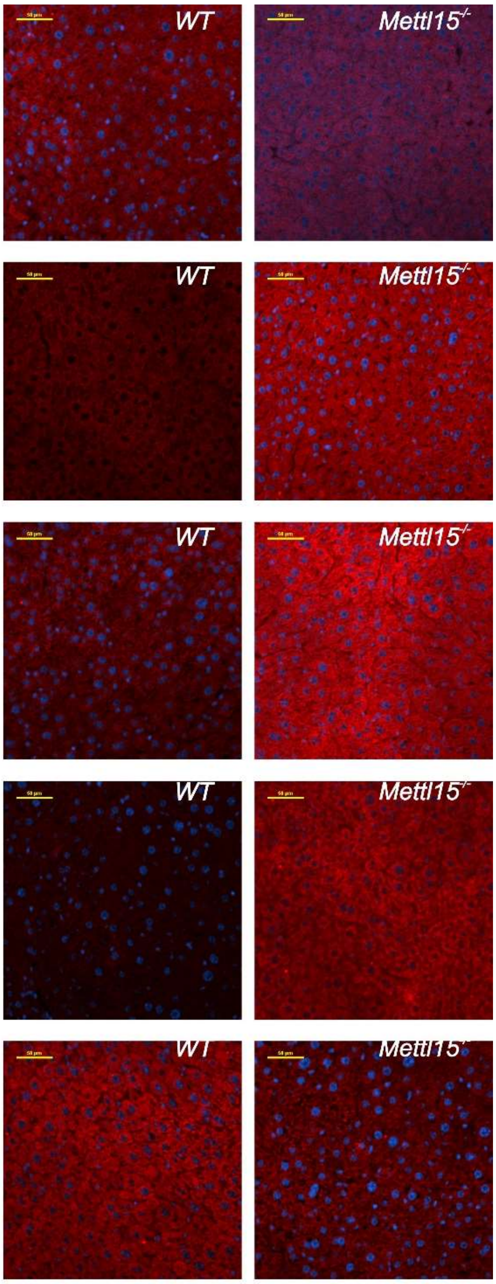
WT
Mettl15-/-
WT
Mettl15-/-
WT
Mettl15-/-
WT
Mettl15-/-
WT
Mettl15-/-

(d)

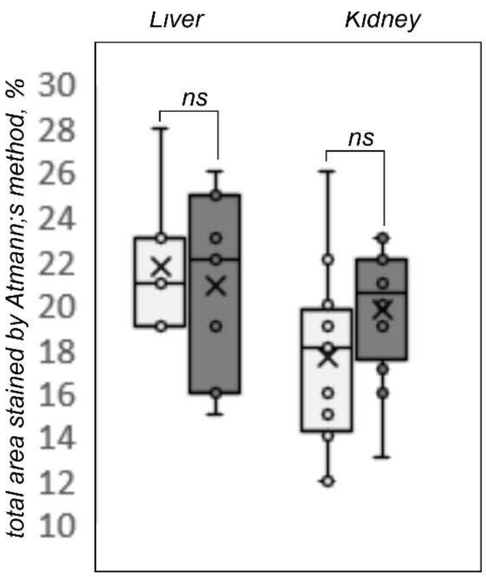

(e)

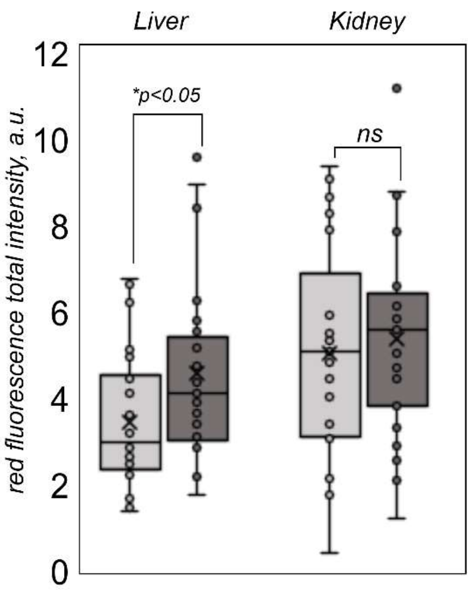

(f)

Supplementary Figure S2. Staining of mitochondria in the wild type and *Mettl15*$^{-/-}$ knockout mice. (**a**) Staining of kidney mitochondria according to Altmann (red color) the wild type (n=12) and *Mettl15*$^{-/-}$ knockout (n=11) mice. (**b**) Staining of liver mitochondria according to Altmann (red color) the wild type (n=7) and *Mettl15*$^{-/-}$ knockout (n=7) mice. (**c**) Staining of kidney mitochondria with anti-VDAC1 antibody (red color) the wild type (n=5) and *Mettl15*$^{-/-}$ knockout (n=5) mice. (**d**) Staining of liver mitochondria with anti-VDAC1 antibody (red color) the wild type (n=5) and *Mettl15*$^{-/-}$ knockout (n=5) mice. (**e**) Quantitation of mitochondria stained by Altmann's method in the liver (left group of bars) and kidney (right group of bars) samples. Wild type (left box plot, light grey, n=7 liver and n=12, kidney) and *Mettl15*$^{-/-}$ knockout (right box plot, dark grey, n=7 liver and n=11, kidney) mice. (**f**) Quantitation of mitochondria stained by anti-VDAC1 antibodies in the liver (left group of bars) and kidney (right group of bars) samples. Wild type (left box plot, light grey, n=5, 15 fields of view, liver and n=5, 15 fields of view, kidney) and *Mettl15*$^{-/-}$ knockout (right box plot, dark grey, n=5, 15 fields of view, liver and n=5, 15 fields of view, kidney) mice.

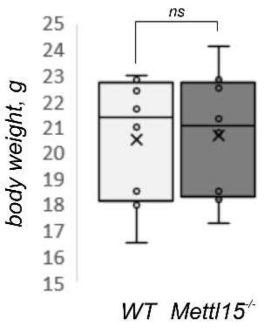

(**a**)

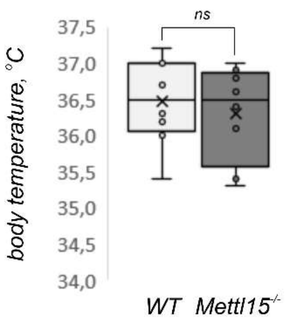

(**b**)

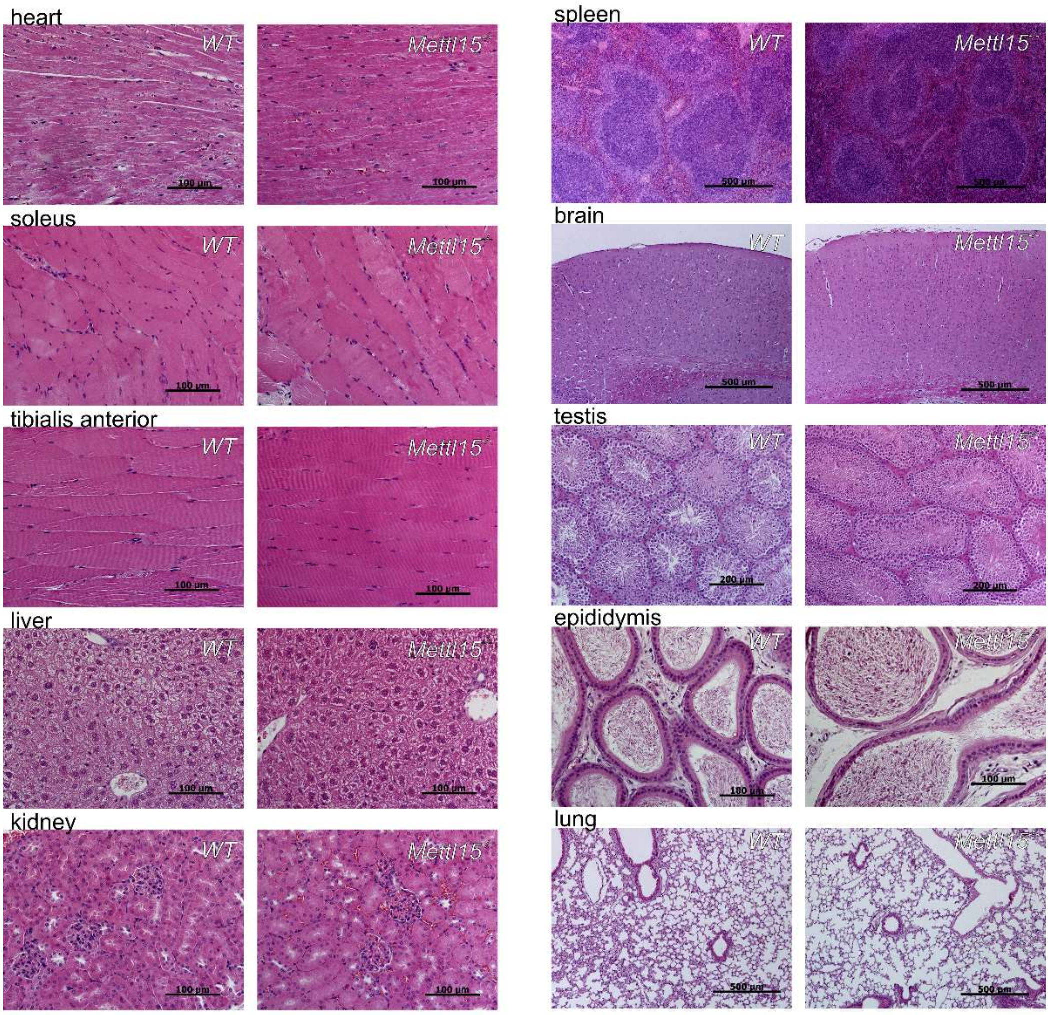

(**c**)

Supplementary Figure S3. General examination of *Mettl15*$^{-/-}$ knockout mice. (**a**) Body weight of the 2 months old wild type (left box plot, light grey, n=8) and *Mettl15*$^{-/-}$ knockout (right box plot, dark grey, n=8) male mice. (**b**) Body temperature of the 2 months old wild type (left box plot, light grey, n=8) and *Mettl15*$^{-/-}$ knockout (right box plot, dark grey, n=8) male mice. (**c**) Histopathology of the wild type (left panels) and *Mettl15*$^{-/-}$ knockout (right panels) mice. Hematoxylin/eosin staining used throughout the panels. Tissue types marked above the panels.

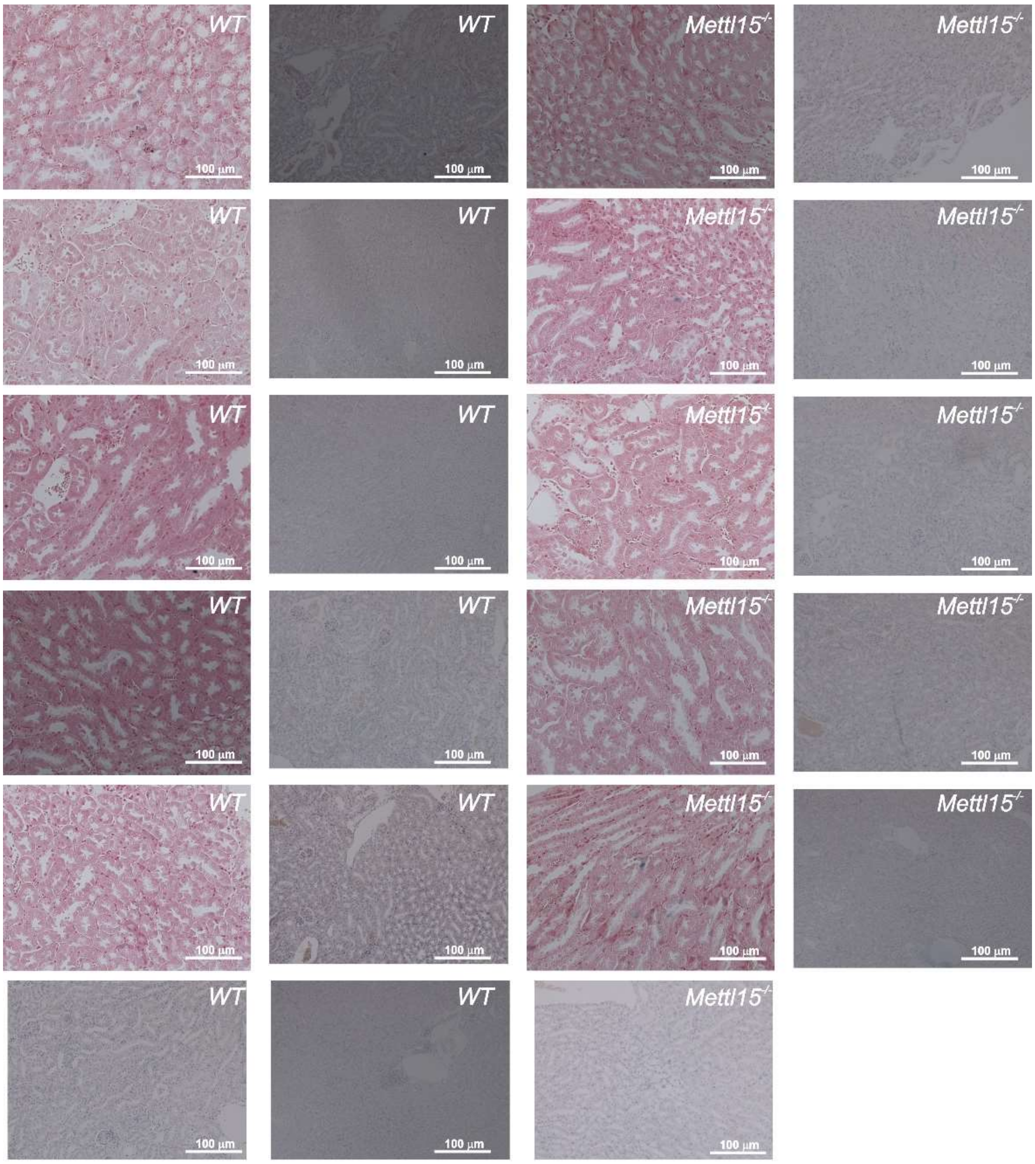

(**a**)

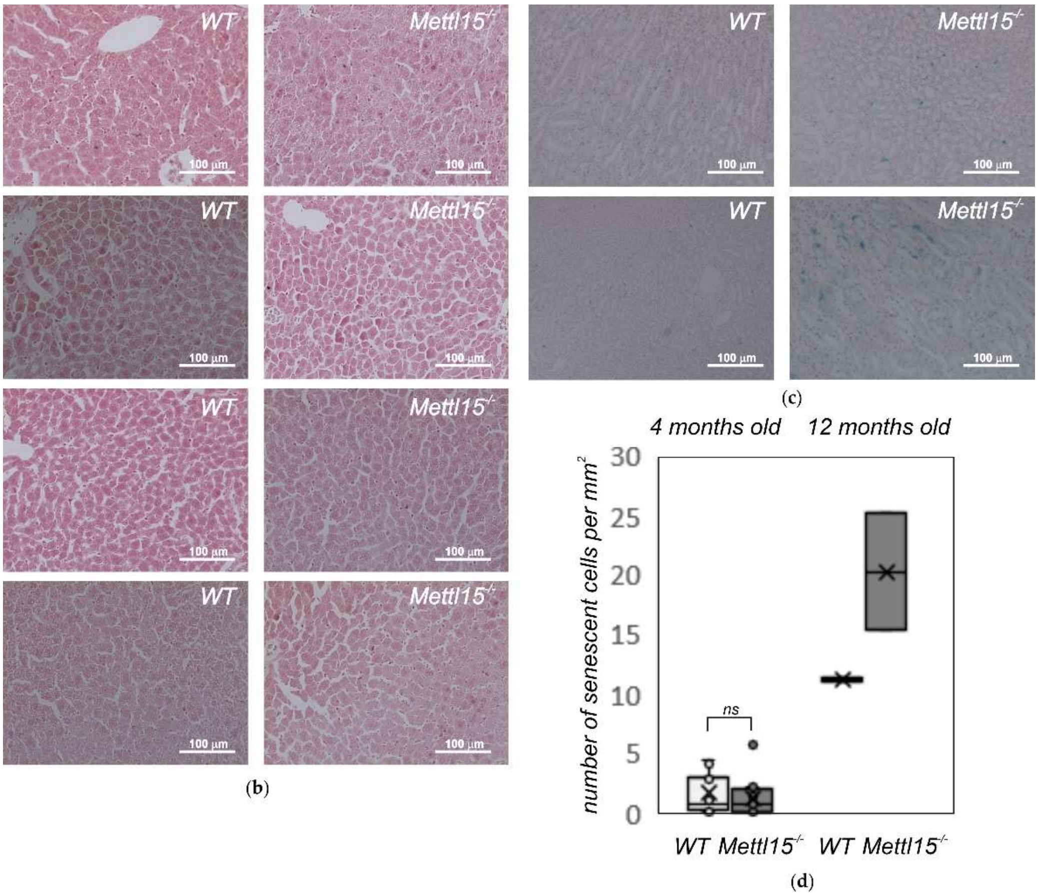

Supplementary Figure S4. Senescent cells (blue) as assessed by senescence associated b-galactosidase staining of the wild type (left panels) and *Mettl15*$^{-/-}$ knockout (right panels) mice. (**a**) Staining of kidney of 4 months old mice (left group of panels, WT, n=12; right group of panels, *Mettl15*$^{-/-}$, n=11). (**b**) Staining of liver of 4 months old mice (left group of panels, WT, n=4; right group of panels, *Mettl15*$^{-/-}$, n=4). (**c**) Staining of kidney of 12 months old mice (left group of panels, WT, n=2; right group of panels, *Mettl15*$^{-/-}$, n=2). (**d**) Quantitation of senescent cells number per mm2 in the kidney of 4 months old mice (left group of bars: WT, n=12; *Mettl15*$^{-/-}$, n=11) and 12 months old mice (right group of bars: WT, n=2; *Mettl15*$^{-/-}$, n=2).

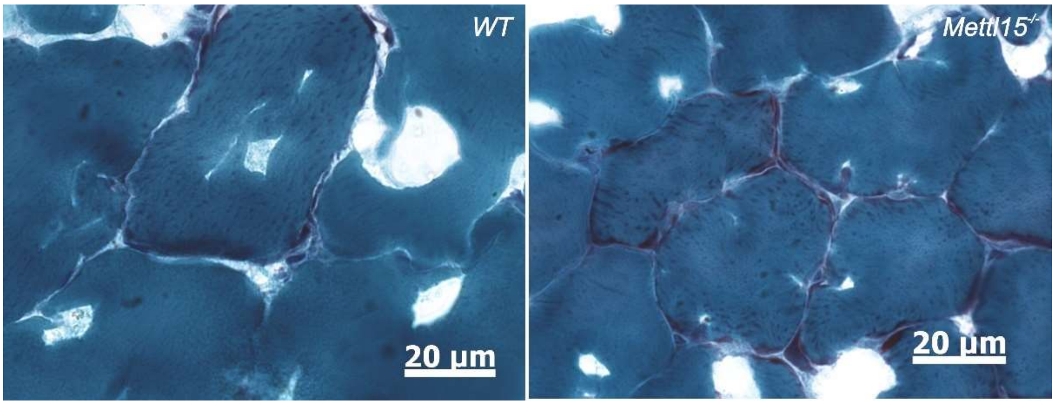

Supplementary Figure S5. Assessment of damaged mitochondria in the skeletal muscle (tibialis anterior). Gomori trichrome staining of the wild type (left panel) and *Mettl15*$^{-/-}$ knockout (right panel). Damaged mitochondria would be stained red.

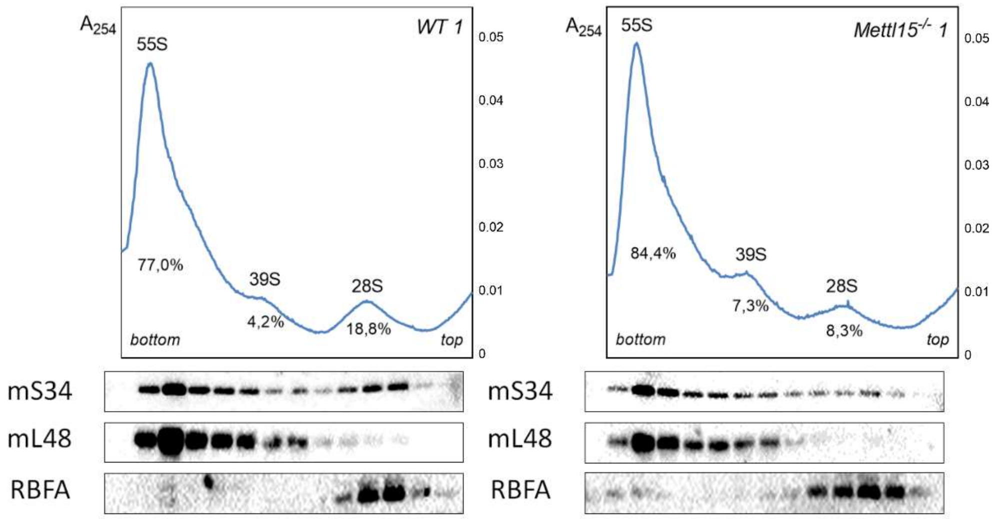
A254
55S
WT 1
0.05
0.04
0.03
0.02
0.01
0
77,0%
39S
4,2%
28S
18,8%
bottom
top
Mettl15-/- 1
84,4%
7,3%
8,3%
mS34
mL48
RBFA

(**a**)

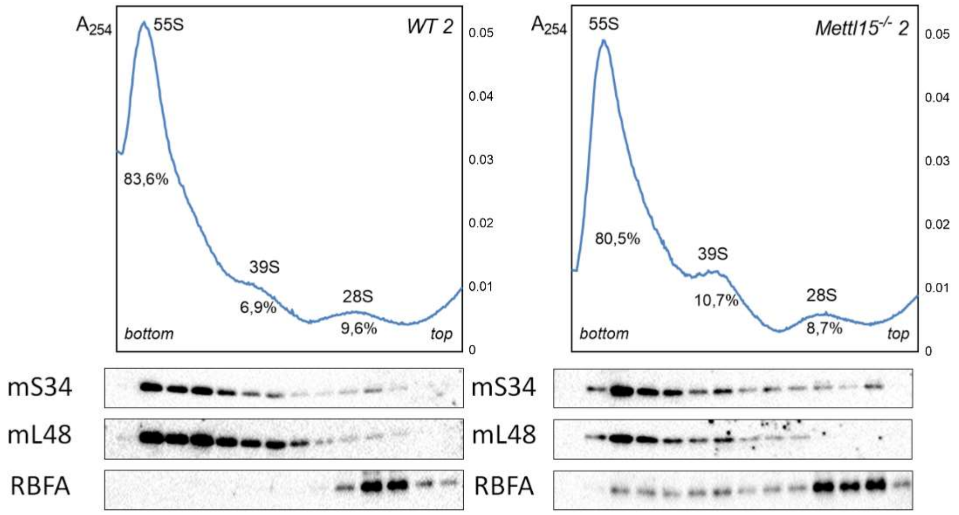
A254
55S
WT 2
0.05
0.04
0.03
0.02
0.01
0
83,6%
39S
6,9%
28S
9,6%
bottom
top
Mettl15-/- 2
80,5%
10,7%
8,7%
mS34
mL48
RBFA

(**b**)

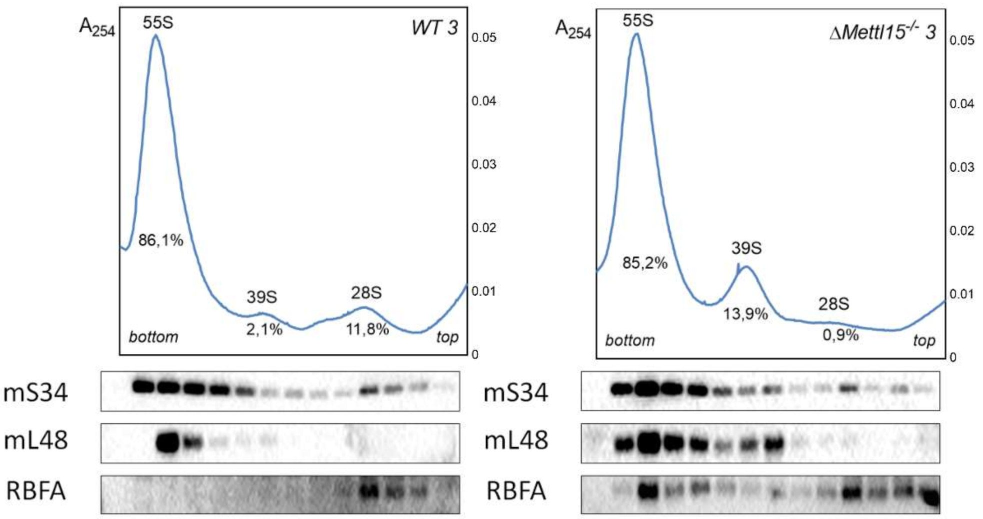
A254
55S
WT 3
0.05
0.04
0.03
0.02
0.01
0
86,1%
39S
2,1%
28S
11,8%
bottom
top
ΔMettl15-/- 3
85,2%
13,9%
0,9%
mS34
mL48
RBFA

(**c**)

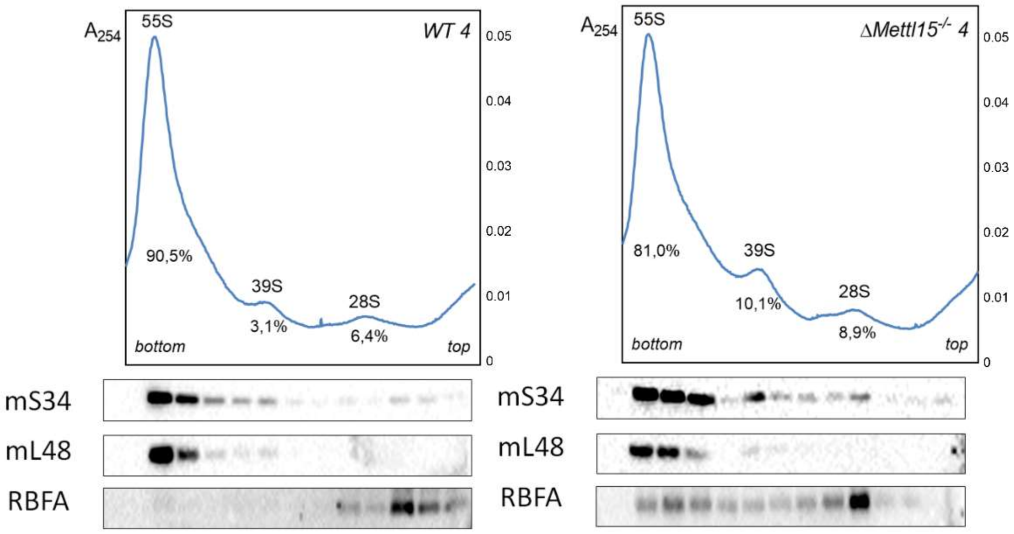
A254
55S
WT 4
90,5%
39S
3,1%
28S
6,4%
bottom
top
0.05
0.04
0.03
0.02
0.01
0
ΔMettl15-/- 4
81,0%
39S
10,1%
28S
8,9%
mS34
mL48
RBFA

(**d**)

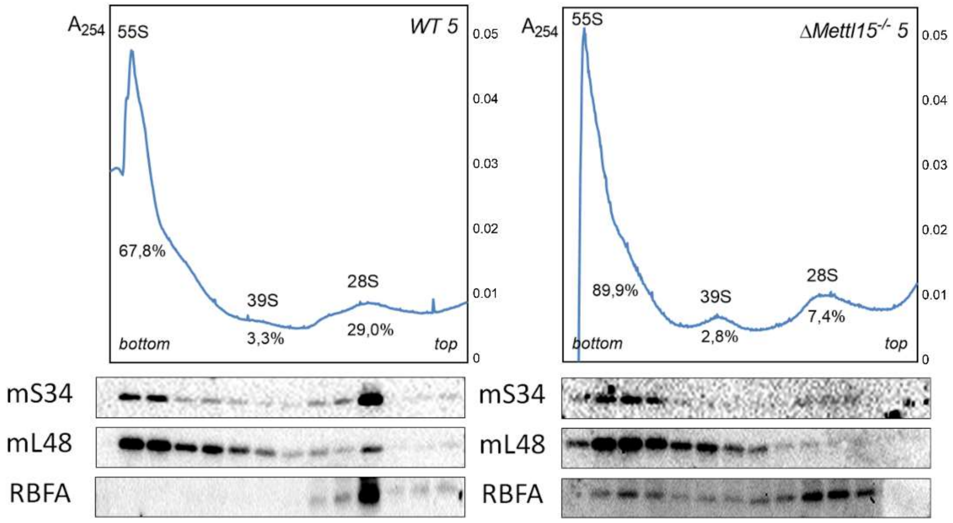
A254
55S
WT 5
67,8%
39S
3,3%
28S
29,0%
bottom
top
0.05
0.04
0.03
0.02
0.01
0
ΔMettl15-/- 5
89,9%
39S
2,8%
28S
7,4%
mS34
mL48
RBFA

(**e**)

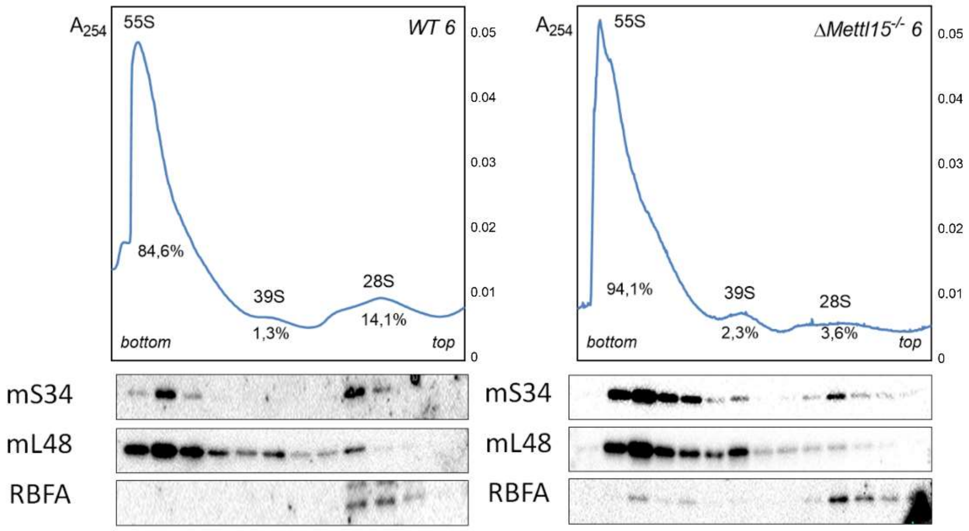
A254
55S
WT 6
84,6%
39S
1,3%
28S
14,1%
bottom
top
0.05
0.04
0.03
0.02
0.01
0
ΔMettl15-/- 6
94,1%
39S
2,3%
28S
3,6%
mS34
mL48
RBFA

(**f**)

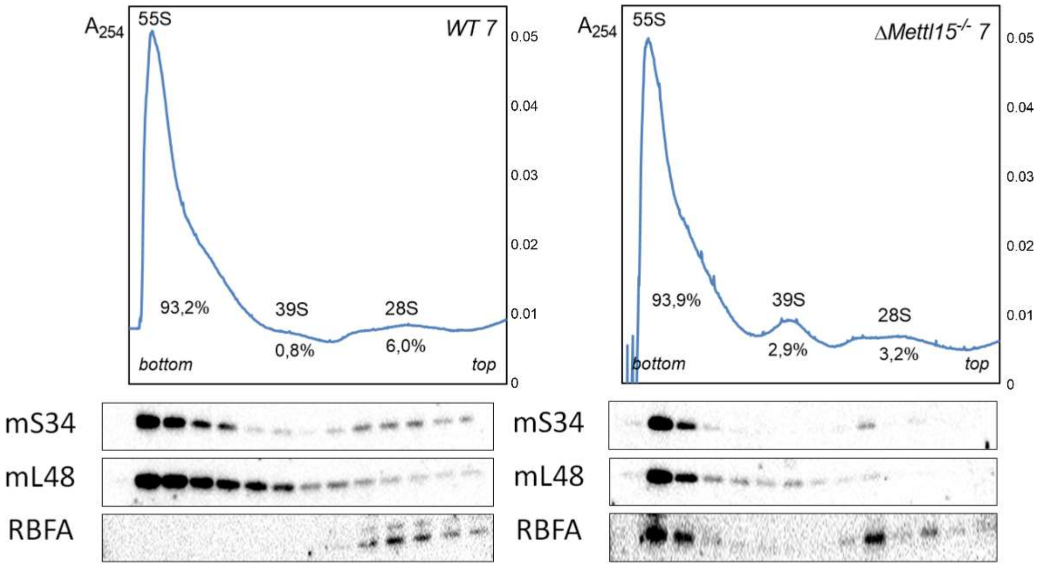

(**g**)

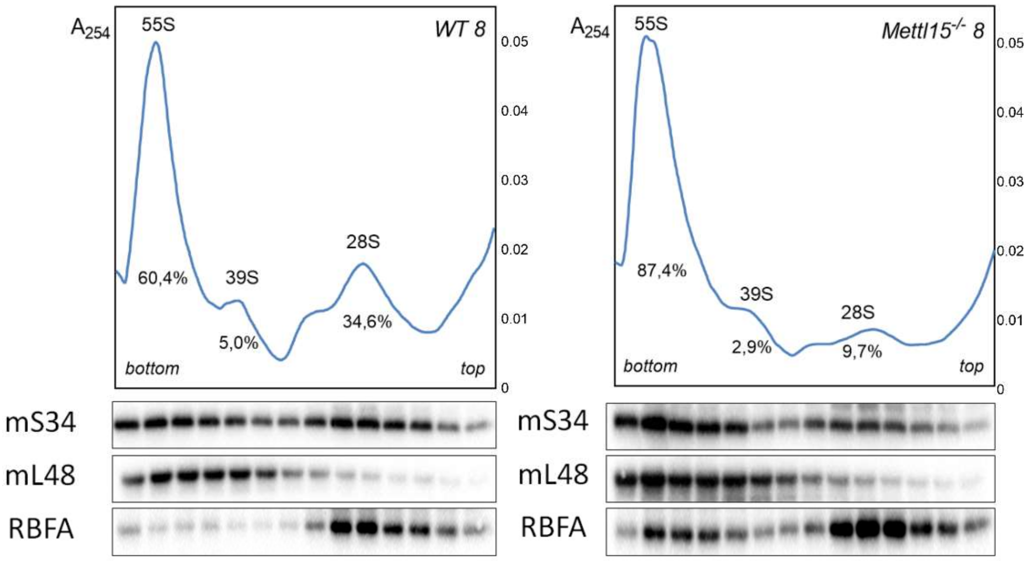

(**h**)

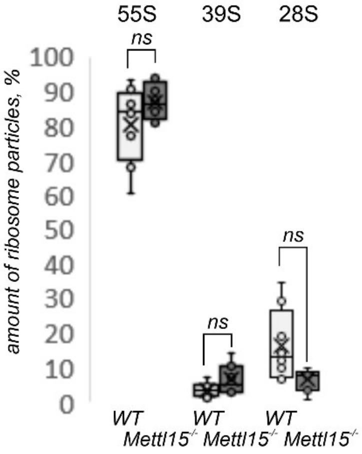

(**i**)

Supplementary Figure S6. Sucrose density gradient profiles of the liver mitochondrial extracts for 365 the wild type (left panels, n=8) *and Mettl15*$^{-/-}$ knockout (right panels, n=8) mice. (**a-h**) A260 is plot- 366.

ted on the upper panes, while immunoblotting of the fractions at the lower panels. Antibodies used are indicated next to the panels. (**i**) Graph summarizing quantitation of the sucrose gradient fractions of the 55S, 39S and 28S particles from liver mitochondria of the wild type (left box plot, light grey, n=8) and *Mettl15*$^{-/-}$ knockout (right box plot, dark grey, n=8) mice.

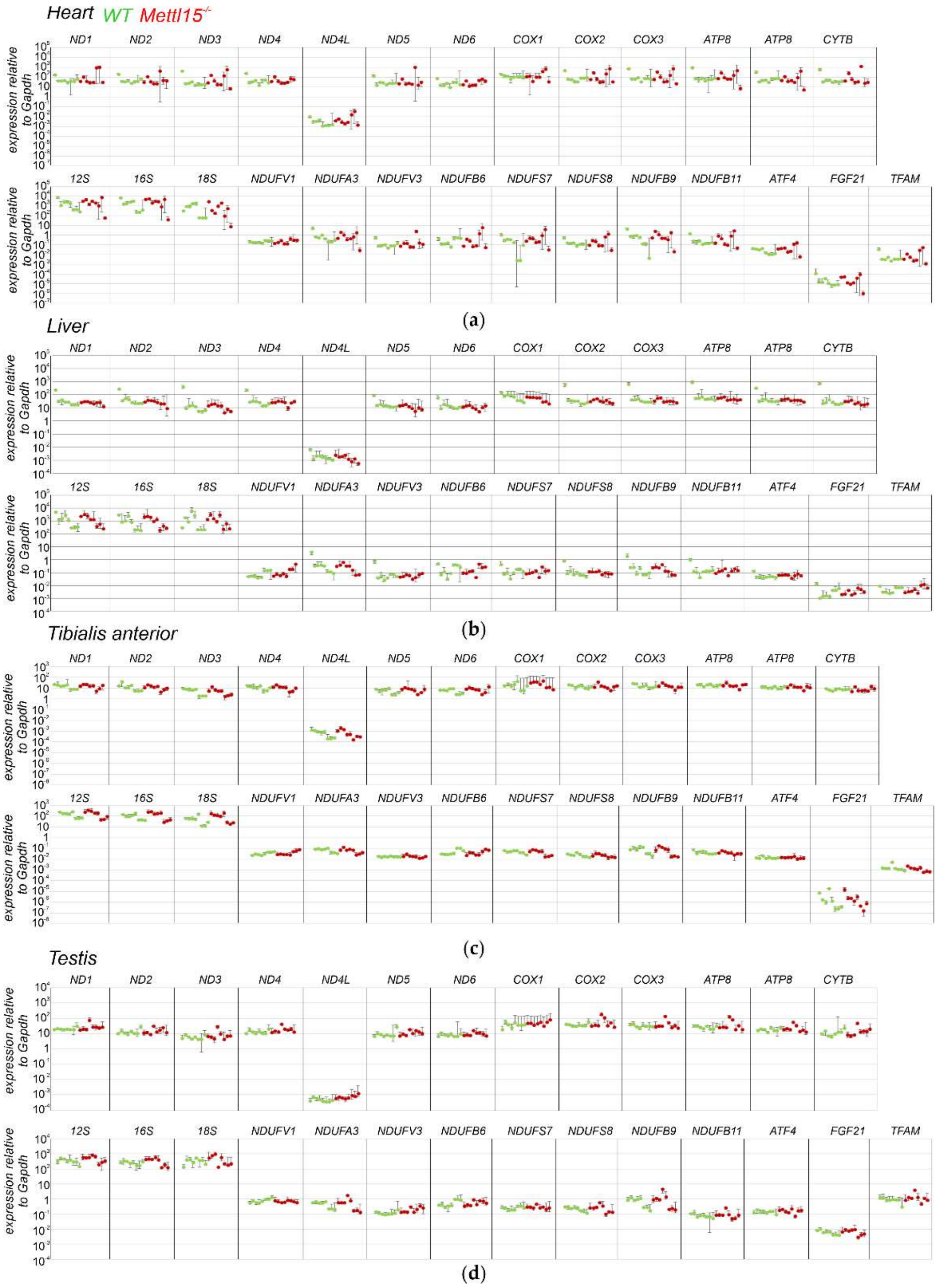

Supplementary Figure S7. Influence of *Mettl15* gene inactivation on gene expression in mice. RT qPCR quantification of the cytosolic and mitochondrial ribosomal RNA, mitochondrial mRNAs and mRNAs of nuclear-encoded mitochondrial proteins as well as mRNAs reported to be overexpressed upon other mitochondrial deficiencies (designations shown above the lanes). Shown are values normalized to the level of *Gapdh* mRNA. Within the lane each point corresponds to a wild type (green dots, n=8) or *Mettl15*$^{-/-}$ knockout (red dots, n=8) animal (i.e. biological replicates). Error bars are used to illustrate the difference between technical replicates. RNA was extracted form heart (**a**), liver (**b**), glycolytic muscle tibialis anterior (**c**) and testis (**d**).

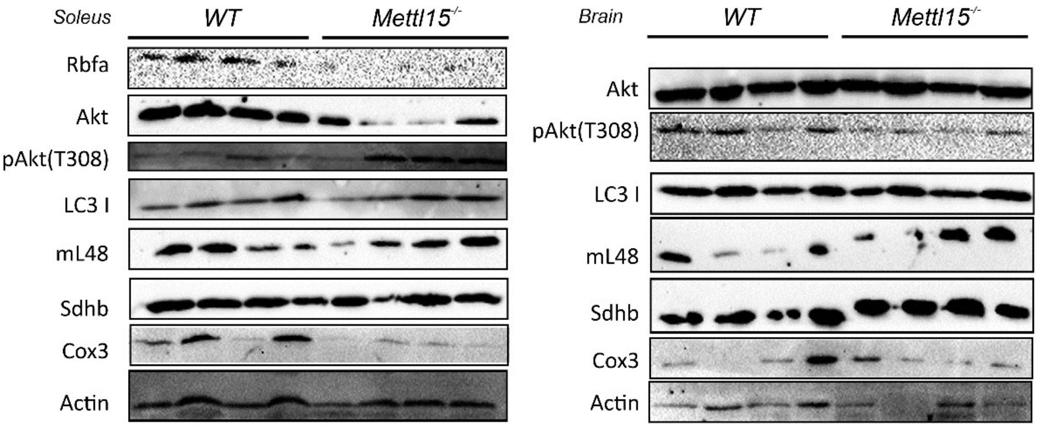

(**a**) (**b**)

Supplementary Figure S8. Influence of *Mettl15* gene inactivation on the amounts of proteins involved in mitochondrial biogenesis and function in mice. (**a**) Immunoblotting of the wild type (left lanes) and *Mettl15*$^{-/-}$ knockout (right lanes) extracts form oxidative skeletal muscle (soleus). Antibodies used are indicated next to the panels. (**b**) Immunoblotting of the wild and *Mettl15*$^{-/-}$ knockout extracts form brain. Designations similar to that of panel (**a**) This experiment reproduces the one shown on the Figure 2b of the main text. Antibodies used are indicated next to the panels.

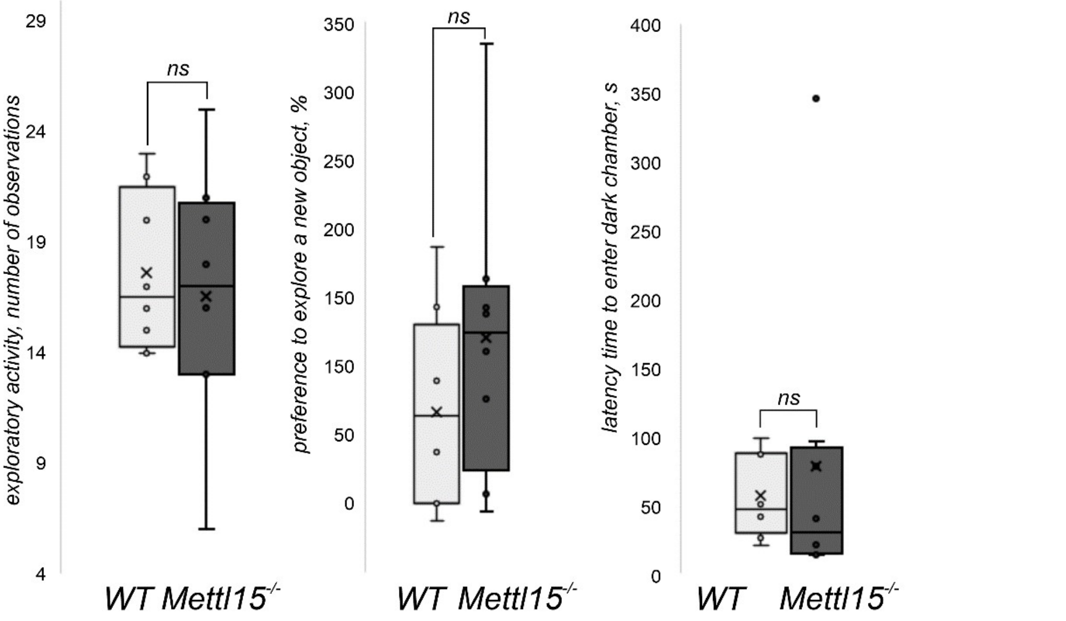

(**a**) (**b**) (**c**)

Supplementary Figure S9. Influence of *Mettl15* gene inactivation on mice behavior. (**a**) Exploratory activity of the wild type (left box plot, light grey, n=8) and *Mettl15*$^{-/-}$ knockout (right box plot, dark grey, n=8) mice measured as a number of observations of objects in an open field test. Interquartile ranges are shown as solid bars, while all data range by thin lines. Horizontal line corresponds to median, while cross to the average. Significance level calculated accordingly to the nonparametric Mann–Whitney U test. (**b**) Exploratory activity of the wild type and *Mettl15*$^{-/-}$ knockout mice measured as a preference (%) to the observation of a new relative to the old object. Designations and number of animals similar to the panel (a). (**c**) Anxiety text in a light-dark box. Shown is the latency time in seconds spent in the illuminated compartment prior to entering the dark compartment. Designations and number of animals similar to the panel (a).

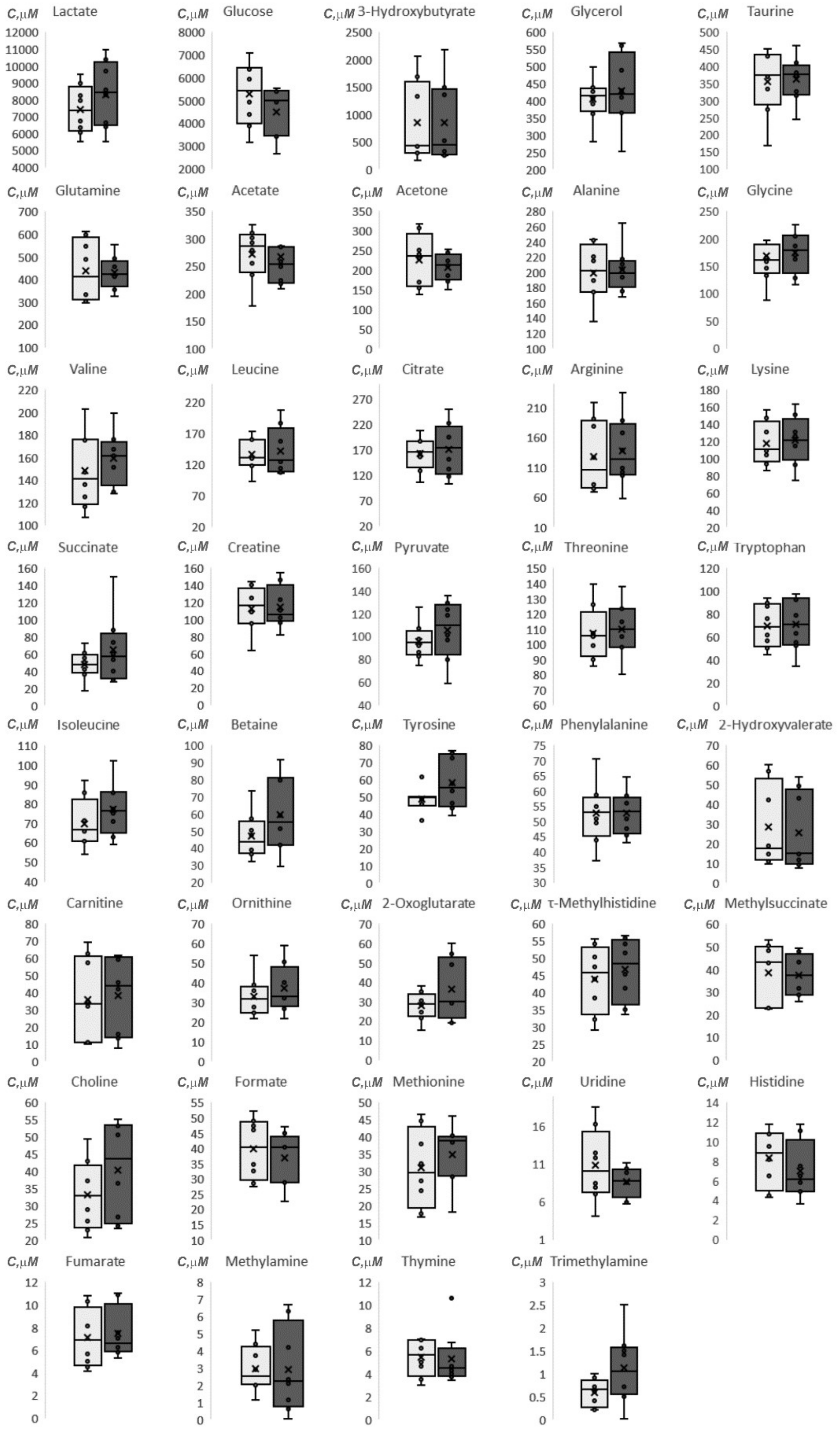

Supplementary Figure S10. Influence of *Mettl15* gene inactivation on plasma metabolite concentration for the mice fed *ad libitum*. Shown are the concentrations (uM) of metabolites designated above the panels as determined by an NMR of plasma. Three mice (biological replicates) were used the wild type cohort (left box plot, light grey, n=8) and three for the *Mettl15*$^{-/-}$ knockout (right box plot, dark grey, n=8). Box plot borders correspond to the maximal and minimal concentrations detected among mice of each cohort, horizontal line to the median while a cross to the average value.

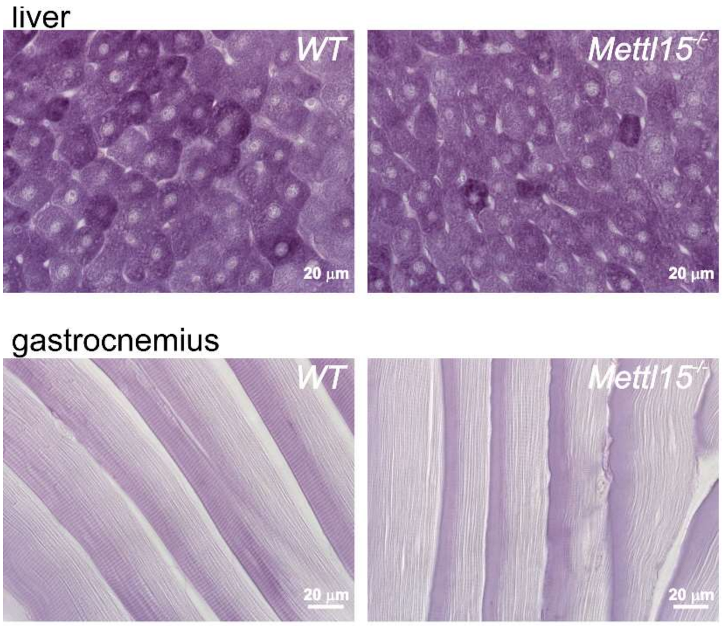

(**a**)

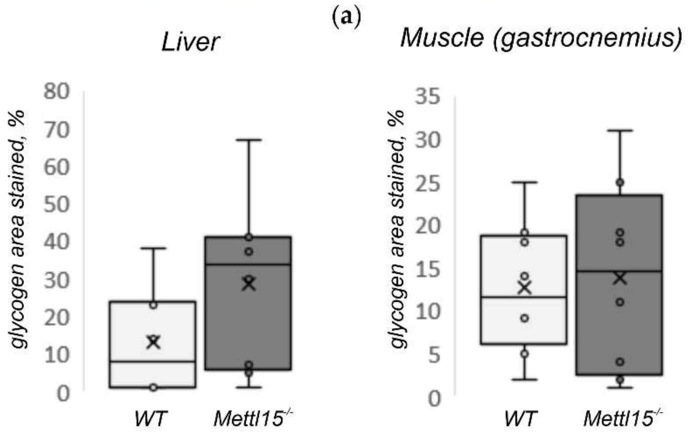

(**b**)

Supplementary Figure S11. Influence of *Mettl15* gene inactivation on glycogen storage. (**a**) Glycogen staining with periodic acid-Schiff stain of the wild type (left panels, n=8) and *Mettl15*$^{-/-}$ knockout (right panels, n=8) liver (upper panels) and muscle (lower panels). Violet color corresponds to glycogen. (**b**) Quantitation of the glycogen amount in the liver (left graphs) and muscle (right graphs). Bars correspond to the wild type (left box plot, light grey, n=8) and *Mettl15*$^{-/-}$ knockout (right box plot, dark grey, n=8) mice.

Supplementary References

1. Laptev, I.; Shvetsova, E.; Levitskii, S.; Serebryakova, M.; Rubtsova, M.; Zgoda, V.; Bogdanov, A.; Kamenski, P.; Sergiev, P.; Dontsova, O. METTL15 Interacts with the Assembly Intermediate of Murine Mitochondrial Small Ribosomal Subunit to Form M4C840 12S RRNA Residue. *Nucleic Acids Res* **2020**, *48*, 8022–8034, doi:10.1093/nar/gkaa522.
2. Ran, F.A.; Hsu, P.D.; Wright, J.; Agarwala, V.; Scott, D.A.; Zhang, F. Genome Engineering Using the CRISPR-Cas9 System. *Nature Protocols* **2013**, *8*, 2281–2308, doi:10.1038/nprot.2013.143.

Averina, O.A.; Vysokikh, M.Y.; Permyakov, O.A.; Sergiev, P.V. Simple Recommendations for Improving Efficiency in Generating Genome-Edited Mice. *Acta Naturae* **2020**, *12*, 42–50, doi:10.32607/actanaturae.10937.

4. Luna, Lee *Histopathologic Methods and Color Atlas of Special Stains and Tissue Artifacts*; American Histolabs, 1993;

5. Lojda, Z.; Gossrau, R.; Schiebler, T.H. *Enzyme Histochemistry: A Laboratory Manual*; Springer-Verlag: Berlin Heidelberg, 1979; ISBN 978-3-540-09269-8.
6. Debacq-Chainiaux, F.; Erusalimsky, J.D.; Campisi, J.; Toussaint, O. Protocols to Detect Senescence-Associated Beta-Galactosidase (SA-Bgal) Activity, a Biomarker of Senescent Cells in Culture and in Vivo. *Nat Protoc* **2009**, *4*, 1798–1806, doi:10.1038/nprot.2009.191.
7. Romeis, Benno *Mikroskopische Technik*; R. Oldenbourg: München, 1948;
8. *Bancroft's Theory and Practice of Histological Techniques*; Suvarna, S.K., Layton, C., Bancroft, J.D., Eds.; Eighth edition.; Elsevier: Amsterdam, 2019; ISBN 978-0-7020-6864-5.
9. Rogers, D.C.; Peters, J.; Martin, J.E.; Ball, S.; Nicholson, S.J.; Witherden, A.S.; Hafezparast, M.; Latcham, J.; Robinson, T.L.; Quilter, C.A.; et al. SHIRPA, a Protocol for Behavioral Assessment: Validation for Longitudinal Study of Neurological Dysfunction in Mice. *Neurosci. Lett.* **2001**, *306*, 89–92, doi:10.1016/s0304-3940(01)01885-7.
10. Huang, C.-C.; Hsu, M.-C.; Huang, W.-C.; Yang, H.-R.; Hou, C.-C. Triterpenoid-Rich Extract from *Antrodia Camphorata* Improves Physical Fatigue and Exercise Performance in Mice. *Evidence-Based Complementary and Alternative Medicine* **2012**, *2012*, 1–8, doi:10.1155/2012/364741.
11. Wang, S.-Y.; Huang, W.-C.; Liu, C.-C.; Wang, M.-F.; Ho, C.-S.; Huang, W.-P.; Hou, C.-C.; Chuang, H.-L.; Huang, C.-C. Pumpkin (Cucurbita Moschata) Fruit Extract Improves Physical Fatigue and Exercise Performance in Mice. *Molecules* **2012**, *17*, 11864–11876, doi:10.3390/molecules171011864.
12. Chen, K.; Li, N.; Fan, F.; Geng, Z.; Zhao, K.; Wang, J.; Zhang, Y.; Tang, C.; Wang, X.; Meng, X. Tibetan Medicine Duoxuekang Capsule Ameliorates High-Altitude Polycythemia Accompanied by Brain Injury. *Front. Pharmacol.* **2021**, *12*, 680636, doi:10.3389/fphar.2021.680636.
13. Li, Q.; Wang, Y.; Cai, G.; Kong, F.; Wang, X.; Liu, Y.; Yang, C.; Wang, D.; Teng, L. Antifatigue Activity of Liquid Cultured *Tricholoma Matsutake* Mycelium Partially via Regulation of Antioxidant Pathway in Mouse. *BioMed Research International* **2015**, *2015*, 1–10, doi:10.1155/2015/562345.
14. Kaur, H.; Kaur, R.; Jaggi, A.S.; Bali, A. Beneficial Role of Central Anticholinergic Agent in Preventing the Development of Symptoms in Mouse Model of Post-Traumatic Stress Disorder. *Journal of Basic and Clinical Physiology and Pharmacology* **2020**, *31*, 20190196, doi:10.1515/jbcpp-2019-0196.
15. Hult, E.M.; Bingaman, M.J.; Swoap, S.J. A Robust Diving Response in the Laboratory Mouse. *J Comp Physiol B* **2019**, *189*, 685–692, doi:10.1007/s00360-019-01237-5.
16. Reger, M.L.; Hovda, D.A.; Giza, C.C. Ontogeny of Rat Recognition Memory Measured by the Novel Object Recognition Task. *Dev. Psychobiol.* **2009**, *51*, 672–678, doi:10.1002/dev.20402.
17. Gaskin, S.; Tardif, M.; Cole, E.; Piterkin, P.; Kayello, L.; Mumby, D.G. Object Familiarization and Novel-Object Preference in Rats. *Behavioural Processes* **2010**, *83*, 61–71, doi:10.1016/j.beproc.2009.10.003.
18. Antunes, M.; Biala, G. The Novel Object Recognition Memory: Neurobiology, Test Procedure, and Its Modifications. *Cogn Process* **2012**, *13*, 93–110, doi:10.1007/s10339-011-0430-z.
19. Eagle, A.; Wang, H.; Robison, A. Sensitive Assessment of Hippocampal Learning Using Temporally Dissociated Passive Avoidance Task. *BIO-PROTOCOL* **2016**, *6*, doi:10.21769/BioProtoc.1821.
20. Xiang, C.; Li, Z.-N.; Huang, T.-Z.; Li, J.-H.; Yang, L.; Wei, J.-K. Threshold for Maximal Electroshock Seizures (MEST) at Three Developmental Stages in Young Mice. 动物学研究 **2019**, *40*, 231–235, doi:10.24272/j.issn.2095-8137.2019.038.
21. Ögren, S.O.; Stiedl, O. Passive Avoidance. In *Encyclopedia of Psychopharmacology*; Stolerman, I.P., Price, L.H., Eds.; Springer Berlin Heidelberg: Berlin, Heidelberg, 2013; pp. 1–10 ISBN 978-3-642-27772-6.
22. Graham, B. Noninvasive, in Vivo Approaches to Evaluating Behavior and Exercise Physiology in Mouse Models of Mitochondrial Disease. *Methods* **2002**, *26*, 364–370, doi:10.1016/S1046-2023(02)00043-9.

23. Serchov, T.; van Calker, D.; Biber, K. Light/Dark Transition Test to Assess Anxiety-like Behavior in Mice. *BIO-PROTOCOL* **016**, *6*, doi:10.21769/BioProtoc.1957.
24. Bourin, M.; Hascoët, M. The Mouse Light/Dark Box Test. *European Journal of Pharmacology* **2003**, *463*, 55–65, doi:10.1016/S0014-2999(03)01274-3.
25. Darvas, M.; Mukherjee, K.; Lee, A.; Ladiges, W. A Novel One-Day Learning Procedure for Mice. *Current Protocols in Mouse Biology* **2020**, *10*, doi:10.1002/cpmo.68.
26. Aibara, S.; Andréll, J.; Singh, V.; Amunts, A. Rapid Isolation of the Mitoribosome from HEK Cells. *J Vis Exp* **2018**, doi:10.3791/57877.
27. Dayal, A.A.; Medvedeva, N.V.; Nekrasova, T.M.; Duhalin, S.D.; Surin, A.K.; Minin, A.A. Desmin Interacts Directly with Mitochondria. *Int J Mol Sci* **2020**, *21*, doi:10.3390/ijms21218122.
28. Reschke, M.; Clohessy, J.G.; Seitzer, N.; Goldstein, D.P.; Breitkopf, S.B.; Schmolze, D.B.; Ala, U.; Asara, J.M.; Beck, A.H.; Pandolfi, P.P. Characterization and Analysis of the Composition and Dynamics of the Mammalian Riboproteome. *Cell Rep* **2013**, *4*, 1276–1287, doi:10.1016/j.celrep.2013.08.014.
29. Golde, W.T.; Gollobin, P.; Rodriguez, L.L. A Rapid, Simple, and Humane Method for Submandibular Bleeding of Mice Using a Lancet. *Lab Anim* **2005**, *34*, 39–43, doi:10.1038/laban1005-39.
30. Diehl, K.H.; Hull, R.; Morton, D.; Pfister, R.; Rabemampianina, Y.; Smith, D.; Vidal, J.M.; van de Vorstenbosch, C.; European Federation of Pharmaceutical Industries Association and European Centre for the Validation of Alternative Methods A Good Practice Guide to the Administration of Substances and Removal of Blood, Including Routes and Volumes. *J Appl Toxicol* **2001**, *21*, 15–23, doi:10.1002/jat.727.
